# Antiplatelet Therapy in Stable Coronary Artery Disease: A Systematic Review and Meta-Analysis

**DOI:** 10.1016/j.jacadv.2026.102633

**Published:** 2026-03-25

**Authors:** Rawan Mohammed Alzahrani, Terad Talmesany, Atheer Atiah Alghamdi, Atheer Fawaz Alhamyani, Danah Abdulaziz Alghamdi, Lama Saleh Alghamdi, Miad Abdullah Alzahrani, Shaima Dhaher Aljohani, Ahmed K. Alsaif, Ahmed Y. Azzam

**Affiliations:** a Faculty of Medicine, Al Baha University, Al Baha, Saudi Arabia; b Head of Department of Family and Community Medicine, Faculty of Medicine, Al Baha University, Al Baha, Saudi Arabia; c College of Nursing, Taif University, Taif, Saudi Arabia; d College of Medicine, Taibah University, Al-Madinah, Saudi Arabia; e College of Medicine, Al-Rayan National Colleges, Al-Madinah, Saudi Arabia; f Director of Clinical Research and Clinical Artificial Intelligence, ASIDE Healthcare, Lewes, Delaware, USA

**Keywords:** coronary artery disease, direct oral anticoagulants, dual antiplatelet therapy, major adverse cardiovascular events, stable coronary artery disease

## Abstract

**Background:**

Stable coronary artery disease (CAD) affects around 200 million people worldwide. Recent evidence with P2Y12 inhibitors, direct oral anticoagulants, and combination strategies has challenged aspirin monotherapy as the standard antithrombotic approach.

**Objectives:**

The purpose of this study was to evaluate antiplatelet strategy efficacy and safety in stable CAD, focusing on monotherapy comparisons, oral anticoagulant strategies in atrial fibrillation with CAD, intensified therapy in high-risk patients, and diabetes-specific strategies.

**Methods:**

We searched databases through May 13, 2025, including randomized controlled trials (RCTs) and observational studies. Primary outcomes were major adverse cardiovascular events (MACEs) and major bleeding. We performed random-effects meta-analyses, network meta-analysis, and meta-regression.

**Results:**

Twenty-three studies (437,662 patients; 13 RCTs, 6 observational, 4 post-hoc) were included. Two RCTs (HOST-EXAM, CAPRIE; n = 24,623) demonstrated clopidogrel superiority over aspirin for MACE (hazard ratio [HR] 0.73 [0.59-0.90]) and bleeding (HR 0.63 [0.41-0.97]). In atrial fibrillation with stable CAD, 2 RCTs (AFIRE, EPIC-CAD; n = 3,276) showed anticoagulant monotherapy had superior efficacy (HR 0.61 [0.49-0.76]) and safety (HR 0.52 [0.36-0.76]) vs combination therapy; observational data (Lamberts; n = 8,700) showed discordant results. Four RCTs showed intensified therapy reduced MACE (HR 0.85 [0.80-0.91]) but increased bleeding (HR 1.85 [1.65-2.07]).

**Conclusions:**

Based on 2 RCTs, clopidogrel monotherapy may offer advantages over aspirin in stable CAD. RCT evidence supports anticoagulant monotherapy in atrial fibrillation with stable CAD beyond 12 months post-revascularization. Intensified strategies may benefit selected high-risk patients though narrow therapeutic margins (number needed to treat 91 vs number needed to harm 85) necessitate careful patient selection.

Stable coronary artery disease (stable CAD) affects around 200 million people from all over the world, making it one of the most prevalent cardiovascular diseases. Despite advances in preventive cardiology, patients with stable CAD face a significant residual risk of recurrent cardiovascular events, with annual rates of major adverse cardiovascular events (MACE) ranging from 1% to 5% depending on risk profile and secondary prevention strategies.^1^ Antiplatelet therapy represents a cornerstone of stable CAD management, with a primary goal of reducing thrombotic complications while minimizing bleeding risk.^2^

For several years and decades, aspirin monotherapy has been the gold standard of antithrombotic management in stable CAD, supported by early trials demonstrating minimal but significant reductions in cardiovascular events.^1^ However, the narrative of antiplatelet therapy has changed significantly, with the introduction of P2Y12 inhibitors, direct oral anticoagulants (DOACs), and various combination strategies challenging the standard aspirin-centric strategy.^3^^,^^4^ Moreover, the increasing recognition of heterogeneity within stable CAD populations, especially regarding comorbidities like diabetes, atrial fibrillation, or chronic kidney disease, has triggered investigation of tailored approaches for specific patient subgroups.^5^

These advancements have created considerable uncertainty. Multiple large randomized controlled trials (RCTs) have recently investigated alternative strategies, including P2Y12 inhibitor monotherapy, various dual antiplatelet therapy (DAPT) regimens, and DOAC-based management strategies.^6^^,^^7^ In addition to that, real-world observational studies have provided important highlights and considerations into the effectiveness and safety of these strategies in practice settings. The resulting evidence is complex and sometimes contradictory, creating challenges for guideline committees and formulating solid and confident evidence-based consensus.^8^, ^9^, ^10^

Current guidelines offer varying recommendations regarding the choice of antiplatelet agent, treatment duration, and management of special populations. The European Society of Cardiology guidelines recommend long-term single antiplatelet therapy (SAPT) for stable CAD (Class I) but acknowledge possible options beyond aspirin monotherapy in selected patients.^11^ Similarly, American guidelines recognize the advancing evidence but maintain aspirin as the default recommendation.^12^ Neither guidelines provide detailed nor focused guidance for increasingly common scenarios such as stable CAD with concomitant atrial fibrillation or diabetes mellitus, where the antithrombotic risk-benefit calculus is significantly important.

Previous meta-analyses have focused on narrow questions, such as DAPT duration after percutaneous coronary intervention or specific drug comparisons.^13^, ^14^, ^15^, ^16^, ^17^, ^18^, ^19^, ^20^, ^21^, ^22^, ^23^ No analysis has integrated the full spectrum of new evidence across the stable CAD populations and treatment strategies. Such an analysis is important not only to resolve contradictions in the literature but also to identify the best achievable management strategies for specific patient subgroups.

In this manner, we aimed to conduct a systematic review and meta-analysis with primary objectives of: 1) compare the efficacy and safety of different antiplatelet monotherapy options; 2) evaluate strategies for patients with stable CAD and atrial fibrillation; 3) assess the benefit-risk profile of intensified antithrombotic therapy in high-risk stable CAD; 4) investigate diabetes-specific antiplatelet strategies; and 5) perform a detailed comparative safety analysis across treatment strategies. From our study, we aim to provide an evidence-based framework for tailoring antiplatelet therapy to individual stable CAD patients based on their specific characteristics and risk profiles.

## Methods

### Eligibility criteria

We included RCTs, prospective and retrospective cohort studies, and post-hoc analyses of RCTs that investigated and studied antiplatelet therapy strategies in adult patients (≥18 years) with stable CAD. Stable CAD was defined as angiographically confirmed CAD, a history of myocardial infarction (MI; over 12 months prior), or a history of coronary revascularization (over 6 months post-percutaneous coronary intervention [PCI] or over 12 months post-coronary artery bypass grafting). Studies had to report at least one efficacy outcome, such as MACE, cardiovascular death, MI, or stroke, or safety outcome (bleeding or adverse events). We excluded studies primarily focused on acute coronary syndromes, those with fewer than 100 participants, and those with follow-up for less than 3 months. We also excluded studies published only as abstracts or in languages other than English.

We included both RCTs and observational studies to balance internal validity with real-world applicability. While RCTs provide the highest level of evidence for treatment efficacy, observational studies offer complementary information on effectiveness in broader populations, long-term outcomes, and rare events. To account for differences in study design, we utilized design-appropriate risk-of-bias tools and conducted sensitivity analyses restricted to RCTs.

### Outcome definitions

Primary efficacy outcomes included MACE, defined as a composite of cardiovascular death, MI, and stroke according to study-specific definitions; all-cause mortality; cardiovascular mortality; and individual components including MI, ischemic stroke, and hemorrhagic stroke. Primary safety outcomes included major bleeding, defined according to study-specific criteria: Bleeding Academic Research Consortium (BARC) types 3 to 5, TIMI major bleeding, International Society on Thrombosis and Haemostasis major bleeding, or Global Utilization of Streptokinase and Tissue Plasminogen Activator for Occluded Coronary Arteries (GUSTO) severe bleeding. We also assessed minor or clinically relevant nonmajor bleeding (BARC type 2, TIMI minor), intracranial hemorrhage, and gastrointestinal bleeding as separate safety endpoints. Secondary outcomes included stent thrombosis (definite or probable), urgent revascularization, net clinical benefit (composite of efficacy and safety outcomes), and nonhemorrhagic adverse events such as dyspnea and bradyarrhythmia.

### Information sources and search strategy

We searched the following literature databases, PubMed, Scopus, Web of Science, Cochrane Library, the Cochrane Central Register of Controlled Trials (CENTRAL), and Google Scholar from inception to May 13, 2025 following Preferred Reporting Items for Systematic reviews and Meta-Analyses (PRISMA) 2020 guidelines;^24^ however, due to the time-sensitive nature of our topic, we did not register our study with PROSPERO, and we consider this as a limitation in our study. The search strategy combined terms related to stable CAD, antiplatelet therapy, and study design. The comprehensive search strategy for MEDLINE was ((“Coronary Artery Disease”[Mesh] OR “coronary artery disease”[tiab] OR “coronary disease”[tiab] OR “coronary heart disease”[tiab] OR “stable angina”[tiab] OR “chronic coronary syndrome”[tiab] OR “stable coronary disease”[tiab] OR “stable CAD”[tiab]) AND (“Platelet Aggregation Inhibitors”[Mesh] OR “antiplatelet”[tiab] OR “anti-platelet”[tiab] OR “aspirin”[tiab] OR “acetylsalicylic acid”[tiab] OR “clopidogrel”[tiab] OR “prasugrel”[tiab] OR “ticagrelor”[tiab] OR “P2Y12”[tiab] OR “thienopyridine”[tiab] OR “DAPT”[tiab] OR “dual antiplatelet”[tiab] OR “single antiplatelet”[tiab] OR “SAPT”[tiab] OR “rivaroxaban”[tiab] OR “apixaban”[tiab] OR “edoxaban”[tiab] OR “Factor Xa Inhibitors”[Mesh] OR “Direct Oral Anticoagulants”[tiab] OR “DOAC”[tiab]) AND (“randomized controlled trial”[pt] OR “controlled clinical trial”[pt] OR “randomized”[tiab] OR “placebo”[tiab] OR “clinical trials as topic”[mesh] OR “randomly”[tiab] OR “trial”[ti] OR “cohort studies”[mesh] OR “cohort”[tiab] OR “observational”[tiab] OR “registry”[tiab])). Similar search strategies were adapted for other databases. In addition to that, we have manually searched reference lists of included studies and relevant review articles to identify additional eligible studies.

### Study selection and data extraction

We have screened the titles and abstracts for possible eligibility, followed by full-text review of preliminary eligible studies. Data extraction was performed to extract the information on study characteristics (design, publication year, funding source), patient characteristics (sample size, age, sex, comorbidities, prior cardiovascular history), interventions (drug types, doses, duration), outcomes (definitions, follow-up duration, events), and results (effect estimates with confidence intervals (CIs)). For studies with multiple publications, we extracted data from the most complete report and supplemented with information from secondary publications when necessary. Authors were contacted if data were missing or unclear, or to inquire about additional datapoints or results in the event that they were not available in the study but could be shared from the authors' records.

### Risk-of-bias assessment

Risk of bias in RCTs was assessed using the Cochrane Risk of Bias 2 (RoB 2) tool, which evaluates bias across 5 domains: randomization process, deviations from intended interventions, missing outcome data, outcome measurement, and selective reporting. For observational studies, we used the Risk of Bias in Non-Randomized Studies of Interventions (ROBINS-I) tool, which assesses bias in confounding, participant selection, classification of interventions, deviations from intended interventions, missing data, outcome measurement, and selective reporting.

### Data synthesis and statistical methods

We performed meta-analyses using random-effects models with the DerSimonian and Laird method to calculate pooled HRs or risk ratios (RRs) with 95% CIs. The primary analyses were stratified by clinical question: 1) antiplatelet monotherapy comparisons; 2) oral anticoagulant (OAC) strategies in atrial fibrillation with stable CAD; 3) intensified antithrombotic therapy in high-risk patients; 4) diabetes-specific strategies; and 5) comparative safety. Heterogeneity was assessed using I^2^ statistics, with values interpreted as follows: <25% indicating low heterogeneity, 25% to 50% indicating moderate heterogeneity, 50% to 75% indicating high heterogeneity, and >75% indicating significant heterogeneity, consistent with Cochrane Handbook recommendations. We explored sources of heterogeneity using subgroup analyses and meta-regression when applicable.

For the network meta-analysis, we used a frequentist approach based on multivariate random-effects meta-analysis. We assessed transitivity by comparing study characteristics across treatment comparisons and evaluated consistency between direct and indirect evidence using node-splitting methods. Treatments were ranked using surface under the cumulative ranking curve (SUCRA) values for efficacy and safety separately.

We calculated absolute risk differences and numbers needed to treat or harm (NNT/NNH) for main outcomes across different baseline risk categories. The certainty of evidence was assessed using the Grading of Recommendations Assessment, Development and Evaluation (GRADE) approach, which considers risk of bias, inconsistency, indirectness, imprecision, and publication bias.

All statistical analyses were performed using RStudio with R version 4.4.2 (R Foundation for Statistical Computing) with the meta, metafor, and netmeta packages. Statistical significance was set at a 2-sided *P* value < 0.05.

## Results

### Study selection and characteristics

**Figure 1:**
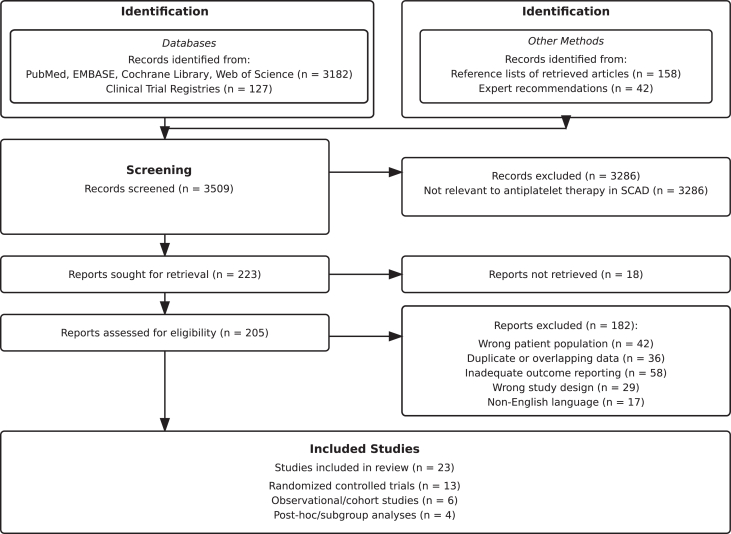
PRISMA Flowchart Diagram

The literature search process has initially identified 3,509 records. After screening and assessment for eligibility, 23 studies met inclusion criteria (Figure 1), in which they included 13 RCTs, 6 observational/cohort studies, and 4 post-hoc/subgroup analyses.^25^, ^26^, ^27^, ^28^, ^29^, ^30^, ^31^, ^32^, ^33^, ^34^, ^35^, ^36^, ^37^, ^38^, ^39^, ^40^, ^41^, ^42^, ^43^, ^44^, ^45^, ^46^, ^47^ The included studies collectively included 437,662 patients with stable CAD. Table 1 presents the characteristics of included studies, showing wide geographic distribution across multiple continents with follow-up durations ranging from 3 months to 8.3 years with a median of 24 months. The study populations included various stable CAD subgroups including post-PCI patients (HOST-EXAM, DAPT Study), diabetic patients with stable CAD (THEMIS, ATHENA-China), patients with concomitant atrial fibrillation (AFIRE, EPIC-CAD, Lamberts), and general stable CAD populations (CAPRIE, COMPASS). Mean age across studies ranged from 59.5 to 91.8 years, with male majority (62.3% to 98.3%). Assessment for risk of bias showed low or some concerns for most RCTs, while observational studies demonstrated moderate to serious risk of bias primarily due to confounding concerns (Supplemental Table 1).

**Table 1:** Characteristics of Included Studies in Meta-Analysis of Antiplatelet Therapy in Stable Coronary Artery Disease

First Author, Year (Study)	Design, Country	Population (N)	Key Inclusion Criteria	Mean/Median Age (years); % Male	Key Comorbidities	Intervention Arms	Primary Outcome Definition	Primary Outcome Results	Major Bleeding Definition	Major Bleeding Results	Follow-up Duration
Öz et al, 2024^25^ (DAPT-TR)	Prospective, multicentric, observational cohort; Turkey	Total: 1,500; stable CAD: 322 CCS	CCS diagnosis (chronic coronary syndrome)	CCS: 62 (56-69); 62.7%	DM: 40.4%; HTN: 71.4%; hyperlipidemia: 62.1%	Fixed-dose DAPT (ASA 75 mg + clopidogrel 75 mg); single arm	Multiple outcomes reported separately	CCS: MI 1.6%, ST 0.6%, TVR 2.5% at 6 months	BARC criteria	BARC 3-5: 0.6% at 6 months	5-6 months
Vallejo-Vaz et al, 2024^26^ (RESRISK)	Retrospective cohort; UK (CPRD & HES)	Total: 294,428; CAD: 266,478	Clinical diagnosis of CAD	71.3 ± 11.2; 62.3%	PVD: 9.7%; HF: 12.7%; CKD: 25.0%; DM: 32.9%	Aspirin monotherapy; single arm	MACE (nonfatal stroke, nonfatal MI, CV death)	92.9 per 1,000 person-years (27.7% cumulative)	Not reported	Not reported	Median 89.9 months
Bian et al, 2024^27^ (ATHENA - China)	Retrospective, observational cohort; China	Total: 13,296; stable CAD: 509 THEMIS-like	Stable CAD >1 mo, specific criteria (pain, prior MI > 3 mo, prior revasc >6 mo)	64.9 ± 10.5; 69.4%	DM: 100%; HTN: 74.3%; anemia: 17.1%	Real-world APMT (mostly DAPT at discharge); single arm	MACE (cardiac death, MI, or stroke)	5.7% at 24 months	BARC type ≥3	1.6% at 24 months	24 months
Cho et al, 2024^28^ (EPIC-CAD)	Multicenter, open-label, adjudicator-masked RCT; South Korea	Total: 1,040; All AF plus stable CAD	STABLE CAD: prev. revasc. (PCI/CABG ≥6 mo) or med managed confirmed CAD	72.1; 77.1%	AF: 100%; DM: ∼40%; HTN: ∼81%; Prior MI: ∼16%	1) Edoxaban Monotherapy (n = 524) 2) Edoxaban + SAPT (n = 516)	Net adverse clinical events: death, MI, stroke, SE, urgent revasc, OR major/CRNM bleed	Edox mono: 6.8% vs Edox + SAPT: 16.2%; HR 0.44 (0.30-0.65), *P* < 0.001	ISTH criteria	Edox mono: 1.3% vs Edox + SAPT: 4.5%; HR 0.32 (0.14-0.73)	12 months
Zou et al, 2023^29^	Retrospective, single-center; China	Total: 351; All elderly stable CAD	Clinical diagnosis of stable CAD, age ≥80 years	91.76 ± 5.01; 98.3%	DLP: 74.4%-80.7%; cancer: 50.0%-58.3%; DM: 47.6%	1) Antiplatelet cessation (n = 211) 2) Antiplatelet continuation (n = 140)	MACE (CV death, nonfatal MI, restenosis, angina)	AP cessation: 73.5% vs AP continuation: 60.0%; HR 1.476 (1.124-1.938), *P* = 0.005	Minor bleeding (clinically important not qualifying as major)	AP cessation: 59.2% vs AP continuation: 54.3%; HR 1.277 (0.950-1.716), *P* = 0.105	Median 98.6 months
Sandner et al, 2022^30^ (TiCAB post-hoc CKD)	Post-hoc analysis of RCT; multicenter	Total: 1843; stable CAD + CKD: 276	Stable CAD or ACS undergoing CABG. CKD: eGFR <60	CKD: ∼74; no CKD: ∼65; CKD: ∼69% male	CKD: 100%; HTN: 89%-94%; DM: 35%-43%	1) Ticagrelor 90 mg BID (n = 123) 2) Aspirin 100 mg QD (n = 153)	MACE (CV death, stroke, MI, or revascularization)	Ticagrelor: 18.2% vs Aspirin: 8.9%; HR 2.15 (1.08-4.30), *P* = 0.03	BARC definition	Ticagrelor: 6.6% vs Aspirin: 4.7%; HR 1.44 (0.52-3.97), *P* = 0.48	1 year
HOST-EXAM (Koo et al, 2021^31^)	Investigator-initiated RCT, open-label; South Korea	Total: 5,438; all post-PCI with DES	Maintained DAPT without events for 6-18 months after PCI with DES	63.5; 74.5%	DM: ∼34.2%; previous MI: ∼16%; PCI for ACS: ∼72%	1) Clopidogrel 75 mg OD (n = 2,710) 2) Aspirin 100 mg OD (n = 2,728)	Composite: All-cause death, nonfatal MI, stroke, readmission due to ACS, BARC bleeding type 3	Clopidogrel: 5.7% vs Aspirin: 7.7%; HR 0.73 (0.59-0.90); *P* = 0.0035	BARC type 3	Clopidogrel: 1.2% vs Aspirin: 2.0%; HR 0.63 (0.41-0.97); *P* = 0.035	24 months
Pracoń et al, 2021^32^ (ISCHEMIA-OMT Poland)	Sub-analysis of RCT; Poland	Total: 5,179; stable CAD: 333	Stable CAD with moderate/severe ischemia	67 (62-75); 70%	DM: 40%; HTN: 88%; prior MI: 32%; prior PCI: 40%	OMT in ISCHEMIA (Poland, n = 264 vs Other countries, n = 3,346)	OMT Goal Attainment (7 goals including aspirin therapy)	Poland: median 6 goals vs Other Countries: median 6 goals; *P* = 0.14	Not reported	Not reported	Median 3.2 years
ASET Pilot Study (Kogame et al, 2020^33^)	Multicenter, single-arm, open-label pilot trial	Total: 201; All stable CAD	PCI for stable CAD with SYNTAX scores <23; successful EES implantation	59.5; 64.7%	DM: 36.8%; stable angina: 94.0%	Aspirin discontinued day of PCI. Prasugrel 60 mg LD post-PCI, then 10 mg OD; single arm	Composite: Cardiac death, spontaneous TV-MI, or definite ST up to 3 months	1 patient (0.5%) - cardiac death	BARC types 3 and 5	1 patient (0.5%) - BARC 5b (fatal ICH)	3 months
THEMIS (Steg et al, 2019^34^)	Randomized, double-blind trial; International	Total: 19,220; All T2DM + stable CAD	Stable CAD (history of PCI/CABG or stenosis ≥50%), T2DM	66; 68.6%	DM: 100%; Prior PCI: 58%; Prior CABG: 21.8%	1) Ticagrelor (60 mg BID) + aspirin (n = 9,619) 2) Placebo + aspirin (n = 9,601)	Composite: CV death, MI, or stroke	Ticagrelor: 7.7% vs Placebo: 8.5%; HR 0.90 (0.81-0.99); *P* = 0.04	TIMI major bleeding	Ticagrelor: 2.2% vs Placebo: 1.0%; HR 2.32 (1.82-2.94); *P* < 0.001	Median 39.9 months
Leiter et al, 2021^35^ (THEMIS-Diabetes Factors)	Post-hoc analyses of RCT; International	THEMIS: 19,220; THEMIS-PCI: 11,154	Same as THEMIS; THEMIS-PCI: with prior PCI	66; 68.6%	DM: 100%; Prior PCI: THEMIS-PCI 100%	Same as THEMIS; THEMIS-PCI subset analysis	Same as THEMIS	THEMIS-PCI: Ticagrelor: 7.3% vs Placebo: 8.6%; HR 0.85 (0.74-0.97); *P* = 0.013	TIMI major bleeding	THEMIS-PCI: Ticagrelor: 2.0% vs Placebo: 1.0%; HR 2.03 (1.48-2.76); *P* < 0.001	Median 39.9 months
Yasuda et al, 2019^36^ (AFIRE)	Multicenter, open-label, randomized trial; Japan	Total: 2,236; mITT: 2215 AF + stable CAD	AF + stable CAD (PCI/CABG >1 y OR angio confirmed CAD)	∼74; ∼79%	AF: 100%; DM: ∼42%; HTN: ∼90%; Prior MI: ∼35%	1) Rivaroxaban monotherapy (n = 1,107) 2) Rivaroxaban + SAPT (n = 1,108)	Stroke, SE, MI, UA requiring revasc, or death from any cause	Riva mono: 4.14%/yr vs Riva + SAPT: 5.75%/yr; HR 0.72 (0.55-0.95)	ISTH criteria (major bleeding)	Riva mono: 1.62%/yr vs Riva + SAPT: 2.76%/yr; HR 0.59 (0.39-0.89), *P* = 0.01	Median 24.1 months (stopped early)
STEEL-PCI (Orme et al, 2018^37^)	Single-center, randomized, open-label, PD study; UK	Total: 180; PCI: 162	Aspirin-treated stable CAD patients planned for elective PCI	65; 82%	DM: 21%; Prior MI: ∼14%; Prior PCI: ∼8%	1) Clopidogrel 600 mg LD/75 mg OD (n = 60) 2) Ticagrelor 180 mg LD/90 mg BID (n = 60) 3) Ticagrelor 180 mg LD/60 mg BID (n = 60)	In vitro adenosine uptake (residual adenosine at 15s)	No difference between groups (*P* = 0.37)	Dyspnea, bleeding events (safety endpoints)	No PLATO major/minor bleeds; dyspnea: T60 7.1% vs T90 19.0% (*P* = 0.09)	1 month
COMPASS (Eikelboom et al, 2017^38^)	Double-blind RCT (3 × 2 partial factorial); International	Total: 27,395; CAD: 90.6%	Stable atherosclerotic vascular disease (CAD or PAD)	68.2; 78%	DM: 37.7%; Prior MI: ∼62%; Prior stroke: 3.8%	1) Rivaroxaban 2.5 mg BID + Aspirin (n = 9,152) 2) Rivaroxaban 5 mg BID (n = 9,117) 3) Aspirin 100 mg OD (n = 9,126)	Composite: CV death, stroke, or MI	Riva + ASA: 4.1% vs ASA: 5.4%; HR 0.76 (0.66-0.86); *P* < 0.001	Modified ISTH major bleeding	Riva + ASA: 3.1% vs ASA: 1.9%; HR 1.70 (1.40-2.05); *P* < 0.001	Mean 23 months (stopped early)
PEGASUS-TIMI 54 (Bonaca et al, 2015^39^)	Randomized, double-blind, placebo-controlled; International	Total: 21,162; All post-MI	MI 1-3 years prior; age ≥50 y; ≥1 high-risk feature	Median 65; ∼76%	DM: ∼32%; Prior PCI: ∼83%; Multivessel CAD: ∼59.5%	1) Ticagrelor 90 mg BID + ASA (n = 7,050) 2) Ticagrelor 60 mg BID + ASA (n = 7,045) 3) Placebo + ASA (n = 7,067)	Composite: CV death, MI, or stroke	T90: 7.85%, T60: 7.77%, Pla: 9.04%; HR vs Pla: 0.85 (*P* = 0.008) and 0.84 (*P* = 0.004)	TIMI major bleeding	T90: 2.60%, T60: 2.30%, Pla: 1.06%; *P* < 0.001 for each vs Pla	Median 33 months
ZEUS (Valgimigli et al, 2015^40^)	Randomized, single-blinded trial; Not specified	Total: 1,606; “Uncertain DES candidates"	Patients qualifying as “uncertain DES candidates" due to high thrombotic/bleeding risk	Median 74; ∼70.5%	DM: ∼26%; Prior MI: ∼24%; ACS presentation: ∼63%	1) Zotarolimus-eluting stent (n = 802) 2) Bare-metal stent (n = 804)	MACE at 1 year (death, MI, or TVR)	ZES: 17.5% vs BMS: 22.1%; HR 0.76 (0.61-0.95); *P* = 0.011	Bleeding (any)	ZES: 11.6% vs BMS: 10.3% (similar rates)	1 year
Bavry et al, 2015^41^ (AJM)	Observational analysis of INVEST cohort; multiple	Total: 22,576; “Non-ischemic": 13,091; “Ischemic": 9,485	Hypertensive patients with stable CAD	“Non-ischemic": 65.4, “Ischemic": 67.1; “Non-ischemic": 40.3%, “Ischemic": 57.9%	All hypertensive	Aspirin use vs no aspirin use	First occurrence of all-cause mortality, nonfatal MI, or nonfatal stroke	“Non-ischemic": ASA HR 1.11 (0.97-1.28); “Ischemic": ASA HR 0.87 (0.77-0.99), *P* = 0.033	Not specifically reported	Not specifically reported	Mean 2.7 years
Larsen et al, 2015^42^ (PLOS ONE)	Cross-sectional study; Denmark	Total: 581; All stable CAD	Stable, high-risk CAD (prior MI, T2DM or both)	64; 79%	Prior MI: 92%; T2DM: 25%; Both: 17%	All received aspirin 75 mg daily	Association between calprotectin and platelet aggregation	Calprotectin positively correlated with multiplate AA-induced aggregation (r = 0.12, *P* = 0.01)	N/A	N/A	Cross-sectional
Lamberts et al, 2014^43^ (Circulation)	Nationwide cohort study; Denmark	Total: 8,700; All AF + stable CAD	AF patients with stable CAD (≥12 months from MI or PCI)	74.2; 62%	Prior MI: ∼64%; Prior PCI: ∼39%; DM: ∼17%	VKA mono; VKA + ASA; VKA + clopidogrel; VKA + DAPT; ASA mono; Clopidogrel mono; ASA + clopidogrel	MI/coronary death; Thromboembolism	VKA + ASA vs VKA mono: HR 1.12 (0.94-1.34); VKA + Clop vs VKA mono: HR 1.53 (0.93-2.52)	Serious bleeding (requiring hospitalization)	VKA + ASA vs VKA mono: HR 1.50 (1.23-1.82); VKA + Clop vs VKA mono: HR 1.84 (1.11-3.06)	Mean 3.3 years
DAPT Study (Mauri et al, 2014^44^)	International, multicenter, RCT; Multiple	Total: 9,961 (DES patients)	Patients post-PCI with DES, completed 12 months of DAPT without events	61.7; ∼75%	DM: ∼30.5%; ACS indication: ∼42.5%; Prior MI: ∼21.5%	1) Continued thienopyridine + ASA for 18 months (n = 5,020) 2) Placebo + ASA for 18 months (n = 4,941)	1. Stent thrombosis 2. MACCE (death, MI, or stroke)	ST: 0.4% vs 1.4%, HR 0.29 (0.17-0.48), *P* < 0.001; MACCE: 4.3% vs 5.9%, HR 0.71 (0.59-0.85), *P* < 0.001	Moderate or severe bleeding (GUSTO)	2.5% vs 1.6%, *P* = 0.001 All-cause mortality: 2.0% vs 1.5%, HR 1.36 (1.00-1.85), *P* = 0.05	18 months treatment (30 months total)
ONSET/OFFSET (Gurbel et al, 2009^45^)	Multicenter, randomized, double-blind; Not specified	Total: 123; All stable CAD	Stable CAD patients taking aspirin (75-100 mg/d)	64; 76%	DM: ∼15%; prior MI: ∼45%; Prior PCI: ∼76%	1) Ticagrelor 180 mg LD/90 mg BID (n = 57) 2) Clopidogrel 600 mg LD/75 mg OD (n = 54) 3) Placebo (n = 12, stopped early)	1. IPA at 2h post-LD 2. IPA slope 4-72h after last dose	Onset (IPA at 2h): Ticagrelor 98% vs Clopidogrel 31% (*P* < 0.0001)	N/A (Pharmacodynamic study)	Dyspnea: Ticagrelor 18.5% vs Clopidogrel 10.0% (*P* < 0.001)	6 weeks
CHARISMA (Bhatt et al, 2006^46^)	Prospective, multicenter, RCT; Multiple	Total: 15,603; ∼78% documented vascular disease	Age ≥45 yrs with multiple atherothrombotic risk factors OR documented vascular disease	Median 64; 70.2%	Vascular disease: ∼78%; Multiple risk factors: ∼21%; DM: ∼42%	1) Clopidogrel 75 mg OD + ASA (n = 7,802) 2) Placebo + ASA (n = 7,801)	Composite: MI, stroke, or CV death	Clop + ASA: 6.8% vs ASA: 7.3%; RR 0.93 (0.83-1.05); *P* = 0.22	Severe bleeding (GUSTO)	Clop + ASA: 1.7% vs ASA: 1.3%; RR 1.25 (0.97-1.61); *P* = 0.09	Median 28 months
CAPRIE (1996)	Randomised, blinded, international trial; Multiple	Total: 19,185; MI: ∼33%	Recent (≤6 mo) ischemic stroke, recent (≤35d) MI, or symptomatic PAD	∼62.5; ∼72%	Stroke: ∼33%; MI: ∼33%; PAD: ∼33%	1) Clopidogrel 75 mg OD (n = 9,599) 2) Aspirin 325 mg OD (n = 9,586)	Composite: Ischemic stroke, MI, or vascular death	Clopidogrel: 5.32%/year vs Aspirin: 5.83%/year; RRR 8.7% (0.3-16.5); *P* = 0.043	Safety profile comparison	Similar profile. ICH: Clop 0.33% vs Asp 0.47%; GI hemorrhage: Clop 0.52% vs Asp 0.72%	Mean 1.91 years

ACS = acute coronary syndrome; AF = atrial fibrillation; AP = antiplatelet; APMT = antiplatelet monotherapy; ASA = acetylsalicylic acid (Aspirin); BARC = Bleeding Academic Research Consortium; BID = twice daily; BMS = bare-metal stent; CABG = coronary artery bypass grafting; CAD = coronary artery disease; CCS = chronic coronary syndrome; CKD = chronic kidney disease; CPRD = Clinical Practice Research Datalink; CRNM = Clinically Relevant Non-Major; CV = cardiovascular; DAPT = dual antiplatelet therapy; DES = drug-eluting stent; DLP = dyslipidemia; DM = diabetes mellitus; EES = everolimus-eluting stent; eGFR = estimated glomerular filtration rate; GI = gastrointestinal; GUSTO = Global Utilization of Streptokinase and Tissue Plasminogen Activator for Occluded Coronary Arteries; HES = Hospital Episode Statistics; HF = heart failure; HR = hazard ratio; HTN = hypertension; ICH = intracranial hemorrhage; IPA = inhibition of platelet aggregation; ISTH = International Society on Thrombosis and Haemostasis; LD = loading dose; MACCE = Major Adverse Cardiac and Cerebrovascular Events; MACE = major adverse cardiovascular events; MI = myocardial infarction; mITT = modified Intention-To-Treat; OD = once daily; OMT = optimal medical therapy; PAD = peripheral artery disease; PCI = percutaneous coronary intervention; PD = pharmacodynamic; PVD = polyvascular disease; RCT = randomized controlled trial; Revasc = revascularization; RR = relative risk; RRR = relative risk reduction; SAPT = single antiplatelet therapy; SE = systemic embolism; ST = stent thrombosis; Stable CAD = stable coronary artery disease; T2DM = type 2 diabetes mellitus; TiCAB = Ticagrelor or Aspirin after Coronary Artery Bypass; TIMI = Thrombolysis In Myocardial Infarction; TV-MI = target vessel myocardial infarction; TVR = target vessel revascularization; UA = unstable angina; VKA = vitamin k antagonist; ZES = zotarolimus-eluting stent.

### Antiplatelet monotherapy comparisons

Table 2 summarizes the comparative effectiveness of antiplatelet monotherapy options. In direct comparison of clopidogrel vs aspirin, 2 RCTs (HOST-EXAM, CAPRIE) consistently demonstrated superior efficacy of clopidogrel for reducing composite endpoints. In the HOST-EXAM trial (n = 5,438), clopidogrel monotherapy significantly reduced the primary composite outcome compared to aspirin (5.7% vs 7.7%; HR 0.73 [95% CI 0.59-0.90]; *P* = 0.0035), with similar benefit observed for thrombotic events (3.7% vs 5.5%; HR 0.68 [0.52-0.87]; *P* = 0.0028). We found that clopidogrel demonstrated significantly lower rates of both major bleeding (BARC type 3: 1.2% vs 2.0%; HR 0.63 [0.41-0.97]; *P* = 0.035) and minor bleeding (BARC type 2: 2.3% vs 3.3%; HR 0.70 [0.51-0.98]; *P* = 0.036). The CAPRIE trial similarly showed lower event rates with clopidogrel (5.32%/year vs 5.83%/year; relative risk reduction 8.7% [0.3-16.5]; *P* = 0.043), with comparable or better safety profile. The single-arm ASET pilot study suggested feasibility of prasugrel monotherapy after PCI in selected low-risk stable CAD patients, with only one adverse event (0.5%) during 3-month follow-up.

**Table 2:** Antiplatelet Monotherapy Comparative Effectiveness

	HOST-EXAM (2021) Clopidogrel vs Aspirin	CAPRIE (1996) Clopidogrel vs Aspirin	Bavry et al. (2015)^41^ Aspirin vs No Aspirin	ASET Pilot (2020) Prasugrel Monotherapy
Study design	RCT, open-label	RCT, blinded	Observational analysis	Single-arm pilot study
Population	Post-PCI with DES (6-18 months after PCI)	Recent MI, stroke, or PAD	Hypertensive stable CAD patients	Stable CAD with EES implantation
Number	5,438 (Clop: 2,710; ASA: 2,728)	19,185 (Clop: 9,599; ASA: 9,586)	22,576 (Prior ischemic: 9,485; No prior ischemic: 13,091)	201
Primary efficacy outcome	Composite: All-cause death, non-fatal MI, stroke, readmission due to ACS, BARC type 3 bleeding	Composite: Ischemic stroke, MI, or vascular death	Composite: All-cause mortality, non-fatal MI, or non-fatal stroke	Composite: Cardiac death, spontaneous TV-MI, or definite stent thrombosis
Primary efficacy results	Clop: 5.7% vs ASA: 7.7% HR 0.73 (0.59-0.90); *P* = 0.0035	Clop: 5.32%/year vs ASA: 5.83%/year RRR 8.7% (0.3-16.5); *P* = 0.043	Prior ischemic: ASA HR 0.87 (0.77-0.99); *P* = 0.033 No prior ischemic: ASA HR 1.11 (0.97-1.28); *P* = 0.13	0.5% (1 event: cardiac death) at 3 months
Thrombotic composite outcome	Cardiac death, MI, ischemic stroke, ACS readmission, definite/probable ST	Not separately reported	Not separately reported	Not applicable
Thrombotic composite results	Clop: 3.7% vs ASA: 5.5% HR 0.68 (0.52-0.87); *P* = 0.0028	Not separately reported	Not separately reported	0.5% (same as primary outcome)
All-cause death	Clop: 1.9% vs ASA: 1.3% HR 1.43 (0.93-2.19); *P* = 0.101	Not separately reported	Prior ischemic: ASA HR 0.79 (0.69-0.90); *P* = 0.0011	0.5% (1 event)
Cardiovascular death	Clop: 0.7% vs ASA: 0.5% HR 1.37 (0.69-2.73); *P* = 0.374	Not separately reported	Not separately reported	0.5% (1 event)
Myocardial infarction	Clop: 0.7% vs ASA: 1.0% HR 0.65 (0.36-1.17); *P* = 0.150	Clop-ASA difference: −0.5%/year *P* < 0.05	Not separately reported	0% (0 events)
Stroke (any)	Clop: 0.7% vs ASA: 1.6% HR 0.42 (0.24-0.73); *P* = 0.002	Not separately reported	Prior ischemic: ASA HR 1.02 (0.75-1.38); *P* = 0.90 No prior ischemic: ASA HR 1.86 (1.22-2.82); *P* = 0.0039	0.5% (1 event - hemorrhagic)
Ischemic stroke	Clop: 0.5% vs ASA: 1.0% HR 0.54 (0.28-1.04); *P* = 0.064	Clop-ASA difference: −0.2%/year *P* < 0.05	Not separately reported	0% (0 events)
Hemorrhagic stroke	Clop: 0.2% vs ASA: 0.6% HR 0.24 (0.08-0.70); *P* = 0.010	Not separately reported	Not separately reported	0.5% (1 event)
Major bleeding	BARC type 3: Clop: 1.2% vs ASA: 2.0% HR 0.63 (0.41-0.97); *P* = 0.035	Not specifically reported ICH: Clop 0.33% vs ASA 0.47% GI hemorrhage: Clop 0.52% vs ASA 0.72%	Not specifically reported	BARC type 3-5: 0.5% (1 event - fatal ICH)
Minor bleeding	BARC type 2: Clop: 2.3% vs ASA: 3.3% HR 0.70 (0.51-0.98); *P* = 0.036	Not specifically reported	Not specifically reported	None reported
Subgroup analysis: Age	≥65 years: HR 0.79 (0.60-1.03); *P* = 0.077 < 65 years: HR 0.66 (0.47-0.92); *P* = 0.015 p-interaction = 0.42	Not reported	Not reported	Not reported
Subgroup analysis: Gender	Male: HR 0.71 (0.56-0.91); *P* = 0.059 Female: HR 0.80 (0.53-1.20); *P* = 0.275 p-interaction = 0.63	Not reported	Not reported	Not reported
Subgroup analysis: Prior MI	Yes: HR 0.74 (0.52-1.06); *P* = 0.096 No: HR 0.73 (0.56-0.94); *P* = 0.016 p-interaction = 0.94	Not reported	Prior ischemic vs No prior ischemic (see above)	Not reported
Key findings	Clopidogrel monotherapy superior to aspirin monotherapy for both thrombotic and bleeding outcomes	Clopidogrel modestly more effective than aspirin for reducing composite CV events, with similar or better safety profile	Aspirin benefit observed only in patients with prior ischemic events; potential harm (stroke) in those without	Aspirin-free prasugrel monotherapy appears feasible in selected low-risk stable CAD after PCI

ACS = acute coronary syndrome; ASA = acetylsalicylic acid (Aspirin); BARC = bleeding academic research consortium; Clop = clopidogrel; DES = drug-eluting stent; EES = everolimus-eluting stent; GI = gastrointestinal; HR = hazard ratio; ICH = intracranial hemorrhage; MI = myocardial infarction; PAD = peripheral artery disease; PCI = percutaneous coronary intervention; RCT = randomized controlled trial; RRR = relative risk reduction; ST = stent thrombosis; Stable CAD = stable coronary artery disease; TV-MI = target vessel myocardial infarction.

### Intensified antithrombotic therapy in high-risk STABLE CAD

**Figure 2:**
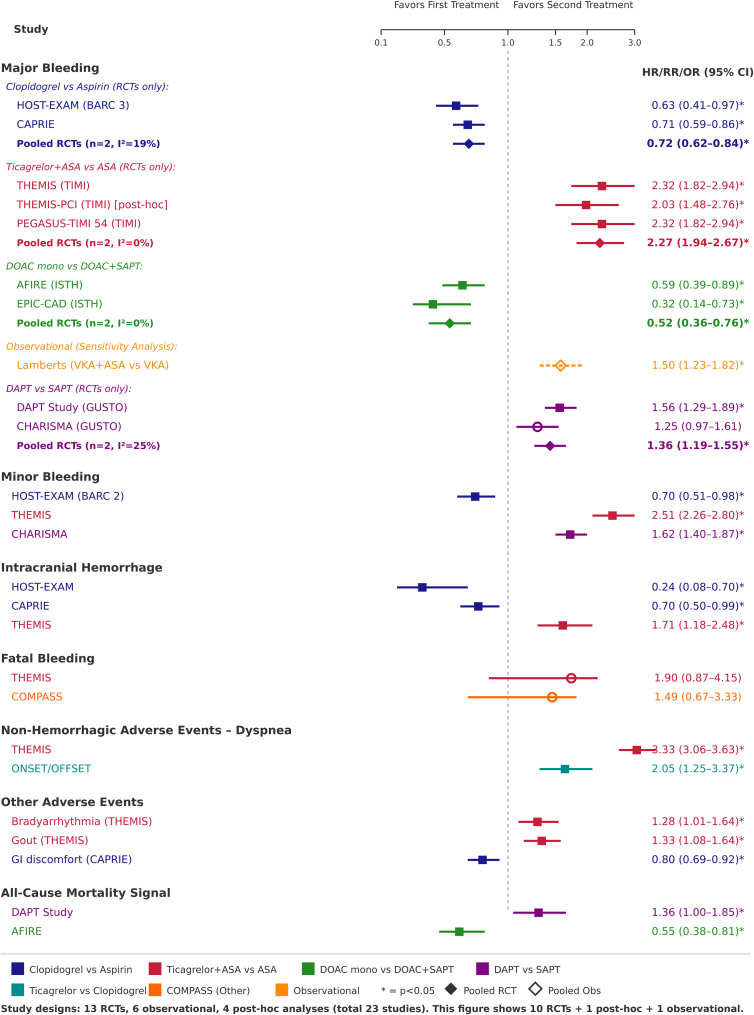
Safety Outcomes Forest Plot Subgrouped

Table 3 presents the estimates of intensified antithrombotic therapy in high-risk stable CAD patients. Across the included trials (COMPASS, PEGASUS-TIMI 54, THEMIS, CHARISMA), intensified therapy demonstrated modest but significant reduction in MACE compared to standard therapy (pooled HR 0.85 [0.80-0.91]; *P* < 0.001). Heterogeneity was moderate (I^2^ = 43%), with individual study HRs ranging from 0.76 (COMPASS) to 0.93 (CHARISMA). This cardiovascular benefit came at the expense of significantly increased major bleeding risk (pooled HR 1.85 [1.65-2.07]; *P* value < 0.001) with significant consistency across trials. In absolute terms, the calculated NNT was 91 (69-133) patients over the median follow-up period, while the NNH was 85 (73-102) for major bleeding, suggesting a neutral to marginally favorable net clinical benefit in unselected high-risk stable CAD populations. Figure 2 presents the forest plot of safety outcomes stratified by treatment comparison, with pooled RCT estimates displayed for each comparison; study designs are clearly indicated, with observational data (Lamberts cohort) shown separately from RCT evidence.

**Table 3:** Meta-Analysis of Intensified Antithrombotic Therapy in High-Risk Stable CAD

Study (Year)	Intervention	Comparator	Primary Outcome	Intervention	Comparator	Effect Size (95% CI)	*P* Value	NNT (95% CI)	Major Bleeding	Intervention	Comparator	Effect Size (95% CI)	*P* Value	NNH (95% CI)
COMPASS (2017)	Rivaroxaban 2.5 mg BID + ASA	ASA alone	CV death, stroke, MI	379/9,152 (4.1%)	496/9,126 (5.4%)	HR 0.76 (0.66-0.86)	<0.001	77 (57-124)	Modified ISTH Major	288/9,152 (3.1%)	170/9,126 (1.9%)	HR 1.70 (1.40-2.05)	<0.001	80 (62-112)
PEGASUS-TIMI 54 (2015) 90 mg	Ticagrelor 90 mg BID + ASA	Placebo + ASA	CV death, MI, stroke	487/7,050 (7.85%)	578/7,067 (9.04%)	HR 0.85 (0.75-0.96)	0.008	84 (54-184)	TIMI Major	127/7,059 (2.60%)	54/7,050 (1.06%)	HR 2.32 (1.68-3.21)	<0.001	65 (50-92)
PEGASUS-TIMI 54 (2015) 60 mg	Ticagrelor 60 mg BID + ASA	Placebo + ASA	CV death, MI, stroke	473/7,045 (7.77%)	578/7,067 (9.04%)	HR 0.84 (0.74-0.95)	0.004	78 (51-167)	TIMI Major	115/7,033 (2.30%)	54/7,050 (1.06%)	HR 2.32 (1.68-3.20)	<0.001	81 (60-125)
THEMIS (2019) Overall	Ticagrelor 60 mg BID + ASA	Placebo + ASA	CV death, MI, stroke	736/9,619 (7.7%)	818/9,601 (8.5%)	HR 0.90 (0.81-0.99)	0.04	125 (65-1,449)	TIMI Major	206/9,562 (2.2%)	100/9,531 (1.0%)	HR 2.32 (1.82-2.94)	<0.001	93 (71-135)
THEMIS-PCI (2019)	Ticagrelor 60 mg BID + ASA	Placebo + ASA	CV death, MI, stroke	404/5,558 (7.3%)	480/5,596 (8.6%)	HR 0.85 (0.74-0.97)	0.013	77 (46-224)	TIMI Major	111/5,558 (2.0%)	57/5,596 (1.0%)	HR 2.03 (1.48-2.76)	<0.001	100 (70-172)
CHARISMA (2006)	Clopidogrel + ASA	Placebo + ASA	CV death, MI, stroke	534/7,802 (6.8%)	573/7,801 (7.3%)	RR 0.93 (0.83-1.05)	0.22	200 (NNT to NNH 84)	GUSTO Severe	130/7,802 (1.7%)	104/7,801 (1.3%)	RR 1.25 (0.97-1.61)	0.09	250 (118-∞)
**Pooled estimate**	Intensified	Standard	Composite CV	**3.01%/year**	**3.55%/year**	**HR 0.85 (0.80-0.91)**	**<0.001**	**91 (69-133)**	Major Bleeding	**1.43%/year**	**0.69%/year**	**HR 1.85 (1.65-2.07)**	**<0.001**	**85 (73-102)**

Values are events/n (%) unless otherwise indicated. **Bold** indicates pooled meta-analytic summary estimates.

ASA = acetylsalicylic acid (Aspirin); CAD = coronary artery disease; CV = cardiovascular; HR = hazard ratio; ISTH = International Society on Thrombosis and Haemostasis; MI = myocardial infarction; NNH = numbers needed harm; NNT = numbers needed to treat; RR = relative risk.

### Treatment strategies by population and clinical outcomes

Table 4 presents NNT/NNH across different treatment strategies and patient populations. In antiplatelet monotherapy comparisons, clopidogrel vs aspirin showed favorable efficacy (NNT 50-196) with comparable or improved safety (NNH for bleeding: no harm to −500, indicating benefit). For OAC strategies in atrial fibrillation with stable CAD patients, OAC monotherapy vs OAC + SAPT demonstrated both superior efficacy (NNT 11-62) and safety (NNH for bleeding: −31 to −88, indicating significantly lower bleeding risk with monotherapy). In intensified therapy regimens for high-risk STABLE CAD, rivaroxaban + acetylsalicylic acid (aspirin) (ASA) vs ASA showed an NNT of 77 with an NNH of 83, while ticagrelor in post-MI patients showed an NNT of 79 with an NNH of 81. The most favorable net clinical benefit profile in the diabetes subgroup was observed in the THEMIS-PCI population (NNT 77, NNH 100). DAPT duration strategies showed greater complexities in benefit-risk assessment, with a significant efficacy benefit for extended DAPT but a concerning mortality signal.

**Table 4:** NNT/NNH by Treatment Strategy and Patient Population

Treatment Comparison	Population/Setting	Event Rate		ARD (%)	NNT (95% CI)	NNH (95% CI)	Time Frame	Net Clinical Benefit
		Treatment	Control					
Antiplatelet monotherapy comparisons:								
Clopidogrel vs Aspirin	Post-PCI (HOST-EXAM)	MACE: 5.7%	MACE: 7.7%	−2.0%	50 (31-121)	-	24 months	Favorable
		Major bleeding: 1.2%	Major bleeding: 2.0%	−0.8%	-	−125 (66-∞)	24 months	
Clopidogrel vs Aspirin	Atherosclerotic disease (CAPRIE)	CV events: 5.32%/yr	CV events: 5.83%/yr	−0.51%/yr	196 (108-1,666)	-	1.91 years	Favorable
		GI bleeding: 0.52%	GI bleeding: 0.72%	−0.20%	-	−500 (N/A)	1.91 years	
OAC Strategies in AF + Stable CAD								
OAC mono vs OAC + SAPT	AF + Stable CAD (EPIC-CAD)	Net clinical events: 6.8%	Net clinical events: 16.2%	−9.4%	11 (8-16)	-	12 months	Strongly favorable
		Major bleeding: 1.3%	Major bleeding: 4.5%	−3.2%	-	−31 (21-61)	12 months	
OAC mono vs OAC + SAPT	AF + Stable CAD (AFIRE)	CV events: 4.14%/yr	CV events: 5.75%/yr	−1.61%/yr	62 (42-180)	-	24.1 months	Favorable
		Major bleeding: 1.62%/yr	Major bleeding: 2.76%/yr	−1.14%/yr	-	−88 (55-211)	24.1 months	
Intensified Therapy in High-Risk Stable CAD								
Riva + ASA vs ASA	Stable CAD/PAD (COMPASS)	MACE: 4.1%	MACE: 5.4%	−1.3%	77 (56-123)	-	23 months	Marginally favorable
		Major bleeding: 3.1%	Major bleeding: 1.9%	+1.2%	-	83 (59-143)	23 months	
Tica 60 mg + ASA vs ASA	Prior MI (PEGASUS)	MACE: 7.77%	MACE: 9.04%	−1.27%	79 (52-167)	-	33 months	Neutral to marginally favorable
		TIMI major: 2.30%	TIMI major: 1.06%	+1.24%	-	81 (60-124)	33 months	
Tica + ASA vs ASA	Diabetes + Stable CAD (THEMIS)	MACE: 7.7%	MACE: 8.5%	−0.8%	125 (70-526)	-	39.9 months	Neutral
		TIMI major: 2.2%	TIMI major: 1.0%	+1.2%	-	83 (65-116)	39.9 months	
Tica + ASA vs ASA	DM + Stable CAD + PCI (THEMIS-PCI)	MACE: 7.3%	MACE: 8.6%	−1.3%	77 (48-181)	-	39.9 months	Favorable in PCI subgroup
		TIMI major: 2.0%	TIMI major: 1.0%	+1.0%	-	100 (72-162)	39.9 months	
Clop + ASA vs ASA	Mixed CV risk (CHARISMA)	MACE: 6.8%	MACE: 7.3%	−0.5%	200 (N/A)	-	28 months	Neutral overall; favorable in symptomatic subgroup
		Severe bleeding: 1.7%	Severe bleeding: 1.3%	+0.4%	-	250 (N/A)	28 months	
DAPT Duration Strategies								
30 mo vs 12 mo DAPT	Post-DES (DAPT Study)	ST: 0.4%	ST: 1.4%	−1.0%	100 (71-166)	-	18 months	Complex; efficacy benefit with mortality signal
		GUSTO mod/sev: 2.5%	GUSTO mod/sev: 1.6%	+0.9%	-	111 (73-233)	18 months	

ARD = absolute risk difference; CI = confidence interval; Clop = clopidogrel; CV = cardiovascular; DAPT = dual antiplatelet therapy; DES = drug-eluting stent; DM = diabetes mellitus; GI = gastrointestinal; MACE = Major Adverse Cardiovascular Events (definitions vary by study); NNH = number needed to harm; NNT = number needed to treat; OAC = oral anticoagulant; PAD = peripheral artery disease; PCI = percutaneous coronary intervention; Riva = rivaroxaban; SAPT = single antiplatelet therapy; ST = stent thrombosis; Stable CAD = stable coronary artery disease; Tica = ticagrelor; TIMI = Thrombolysis in Myocardial Infarction.

### Meta-regression of effect modifiers

Table 5 presents the meta-regression results of factors affecting treatment effect sizes in antiplatelet therapy for stable CAD. For efficacy outcomes, significant moderator variables included publication year (coefficient −0.18 [−0.35 to −0.02] per decade; *P* value = 0.031), mean age (coefficient 0.09 [0.03-0.15] per 5 years; *P* = 0.004), diabetes prevalence (coefficient 0.08 [0.02-0.14] per 10%; *P* = 0.008), and prior MI percentage (coefficient 0.07 [0.01-0.13] per 10%; *P* = 0.017). For safety outcomes, significant moderators included publication year (coefficient 0.22 [0.07-0.38] per decade; *P* = 0.005), follow-up duration (coefficient 0.10 [0.02-0.18] per year; *P* = 0.014), mean age (coefficient 0.11 [0.04-0.18] per 5 years; *P* = 0.002), and treatment characteristics including DAPT vs monotherapy comparison (coefficient 0.31 [0.14-0.48]; *P* < 0.001). Methodological variables including double-blind design and adjudicated endpoints significantly affected efficacy estimates.

**Table 5:** Meta-Regression of Factors Affecting Treatment Effect Sizes in Antiplatelet Therapy for Stable CAD

Moderator Variable	Outcome: MACE/CV Events		Outcome: Major Bleeding	
	Coefficient (95% CI)	*P* Value	Coefficient (95% CI)	*P* Value
Study Design Characteristics				
Publication year (per decade)	−0.18 (−0.35 to −0.02)	0.031	0.22 (0.07-0.38)	0.005
Sample size (per 1,000 participants)	−0.01 (−0.03 to 0.01)	0.412	0.00 (−0.02 to 0.02)	0.887
Follow-up duration (per year)	−0.06 (−0.14 to 0.02)	0.148	0.10 (0.02-0.18)	0.014
RCT vs observational design	−0.15 (−0.28 to −0.02)	0.022	0.09 (−0.06 to 0.24)	0.237
Industry sponsorship	0.02 (−0.12 to 0.17)	0.763	0.12 (−0.04 to 0.28)	0.136
Patient Population Characteristics				
Mean age (per 5 years)	0.09 (0.03-0.15)	0.004	0.11 (0.04-0.18)	0.002
Male percentage (per 10%)	0.04 (−0.06 to 0.14)	0.412	−0.02 (−0.13 to 0.09)	0.697
Diabetes prevalence (per 10%)	0.08 (0.02-0.14)	0.008	0.05 (−0.01 to 0.11)	0.124
Prior MI percentage (per 10%)	0.07 (0.01-0.13)	0.017	0.03 (−0.04 to 0.10)	0.395
Prior PCI percentage (per 10%)	0.05 (−0.01 to 0.11)	0.081	0.02 (−0.05 to 0.09)	0.574
Intervention Characteristics				
P2Y12i vs aspirin comparison	−0.22 (−0.38 to −0.06)	0.007	0.08 (−0.09 to 0.25)	0.358
DAPT vs monotherapy comparison	−0.16 (−0.32 to 0.00)	0.049	0.31 (0.14-0.48)	<0.001
OAC-containing regimen	−0.08 (−0.25 to 0.09)	0.342	0.28 (0.10-0.46)	0.002
Treatment duration (per year)	−0.08 (−0.16 to 0.00)	0.051	0.14 (0.05-0.23)	0.001
Methodological Characteristics				
Adequate allocation concealment	−0.13 (−0.27 to 0.01)	0.066	0.05 (−0.10 to 0.21)	0.510
Double-blind design	−0.19 (−0.34 to −0.04)	0.012	0.16 (0.00-0.32)	0.045
Intention-to-treat analysis	−0.11 (−0.26 to 0.04)	0.154	0.07 (−0.09 to 0.23)	0.383
Adjudicated endpoints	−0.16 (−0.30 to −0.02)	0.028	0.13 (−0.02 to 0.28)	0.089
Outcome Definition Characteristics				
3-point vs expanded MACE definition	0.12 (0.01-0.23)	0.042	—	—
TIMI vs other bleeding criteria	—	—	−0.14 (−0.29 to 0.01)	0.072
ISTH vs other bleeding criteria	—	—	0.09 (−0.07 to 0.25)	0.251
BARC vs other bleeding criteria	—	—	0.02 (−0.15 to 0.19)	0.817
Model Statistics				
Residual I^2^	63.7%		71.8%	
Adjusted R^2^	0.31		0.28	
Number of studies	18		16	

BARC = Bleeding Academic Research Consortium; CI = confidence interval; CV = cardiovascular; DAPT = dual antiplatelet therapy; ISTH = International Society on Thrombosis and Haemostasis; MACE = major adverse cardiovascular events; MI = myocardial infarction; OAC = oral anticoagulant; PCI = percutaneous coronary intervention; P2Y12i = P2Y12 inhibitor; RCT = randomized controlled trial.

### Network meta-analysis results

**Figure 3:**
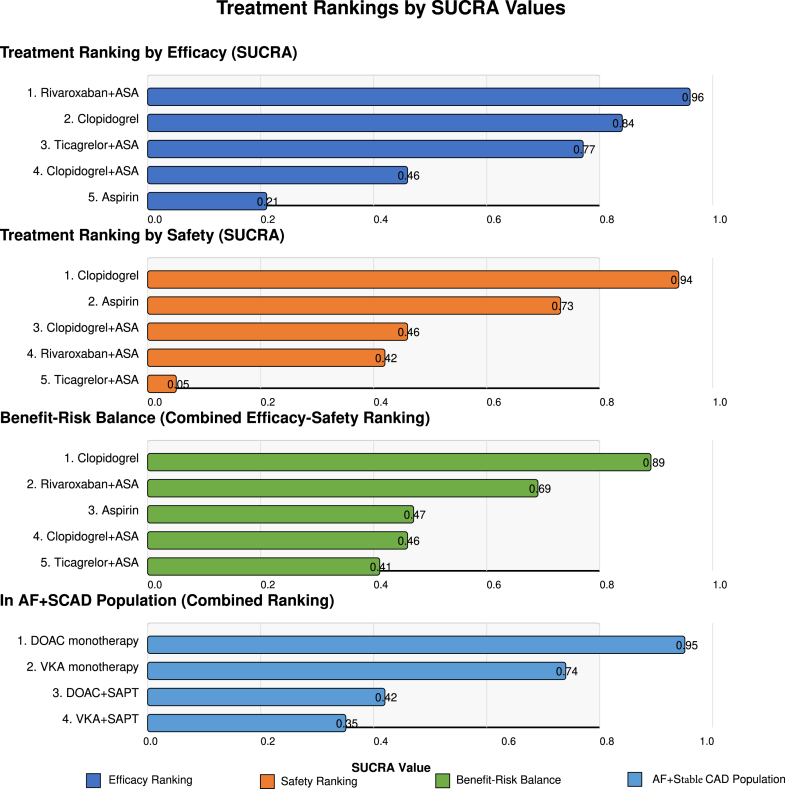
Treatment Rankings According to SUCRA Values

Table 6 summarizes the network meta-analysis of antiplatelet strategies in stable CAD. Direct, indirect, and combined evidence were consistent across most comparisons (consistency *P* values exceeding 0.38), indicating valid network transitivity. For efficacy outcomes, clopidogrel vs aspirin showed significant benefit (combined HR 0.81 [0.73-0.90]), as did ticagrelor + ASA vs aspirin (HR 0.88 [0.82-0.94]). The most significant efficacy benefit was seen with rivaroxaban + ASA vs aspirin (HR 0.76 [0.66-0.86]). In the atrial fibrillation with stable CAD population, DOAC monotherapy vs DOAC + SAPT showed significant benefit (HR 0.69 [0.56-0.85]). For safety outcomes, clopidogrel demonstrated significantly lower bleeding risk vs aspirin (RR 0.72 [0.62-0.84]), while ticagrelor + ASA (RR 2.27 [1.94-2.67]), clopidogrel + ASA (RR 1.36 [1.19-1.55]), and rivaroxaban + ASA (RR 1.70 [1.40-2.05]) all showed significantly increased bleeding compared to aspirin. In the atrial fibrillation with stable CAD population, DOAC monotherapy showed substantially lower bleeding risk than DOAC + SAPT (RR 0.49 [0.36-0.67]). Figure 3 presents treatment rankings by SUCRA values, with clopidogrel ranking highest for the combined efficacy-safety profile (0.89), followed by rivaroxaban + ASA (0.69), aspirin (0.47), clopidogrel + ASA (0.46), and ticagrelor + ASA (0.41).

**Table 6:** Network Meta-Analysis of Antiplatelet Strategies in Stable CAD

Treatment Comparison	Direct Evidence	Indirect Evidence	Combined Evidence	Heterogeneity (I^2^)	Consistency *P* Value
Efficacy Outcome: Major Adverse Cardiovascular Events					
Clopidogrel vs Aspirin	HR 0.78 (0.69-0.89)^a^	HR 0.85 (0.70-1.03)	HR 0.81 (0.73-0.90)^a^	27%	0.41
Ticagrelor + ASA vs Aspirin	HR 0.87 (0.81-0.94)^a^	HR 0.91 (0.77-1.08)	HR 0.88 (0.82-0.94)^a^	11%	0.63
Ticagrelor + ASA vs Clopidogrel + ASA	HR 0.94 (0.83-1.07)	HR 0.87 (0.71-1.06)	HR 0.92 (0.83-1.02)	38%	0.49
Clopidogrel + ASA vs Aspirin	HR 0.93 (0.83-1.05)	HR 0.90 (0.78-1.04)	HR 0.92 (0.84-1.01)	0%	0.71
Rivaroxaban + ASA vs Aspirin	HR 0.76 (0.66-0.86)^a^	-	HR 0.76 (0.66-0.86)^a^	N/A	N/A
Efficacy in AF + Stable CAD Population					
DOAC mono vs DOAC + SAPT	HR 0.69 (0.56-0.85)^a^	-	HR 0.69 (0.56-0.85)^a^	0%	N/A
VKA + ASA vs VKA mono	HR 1.12 (0.94-1.34)	-	HR 1.12 (0.94-1.34)	N/A	N/A
Safety Outcome: Major Bleeding					
Clopidogrel vs Aspirin	RR 0.69 (0.56-0.84)^a^	RR 0.78 (0.61-0.98)^a^	RR 0.72 (0.62-0.84)^a^	19%	0.38
Ticagrelor + ASA vs Aspirin	RR 2.21 (1.84-2.65)^a^	RR 2.45 (1.76-3.41)^a^	RR 2.27 (1.94-2.67)^a^	0%	0.57
Clopidogrel + ASA vs Aspirin	RR 1.41 (1.18-1.67)^a^	RR 1.29 (1.05-1.59)^a^	RR 1.36 (1.19-1.55)^a^	25%	0.44
Rivaroxaban + ASA vs Aspirin	RR 1.70 (1.40-2.05)^a^	-	RR 1.70 (1.40-2.05)^a^	N/A	N/A
Safety in AF + Stable CAD Population					
DOAC mono vs DOAC + SAPT	RR 0.49 (0.36-0.67)^a^	-	RR 0.49 (0.36-0.67)^a^	32%	N/A
VKA + ASA vs VKA mono	HR 1.50 (1.23-1.82)^a^	-	HR 1.50 (1.23-1.82)^a^	N/A	N/A

AF = atrial fibrillation; ASA = acetylsalicylic acid (Aspirin); SAPT = single antiplatelet therapy; DOAC = direct oral anticoagulant; HR = hazard ratio; N/A = not applicable; RR = relative risk; Stable CAD = stable coronary artery disease; SUCRA = Surface Under the Cumulative Ranking Curve (higher values indicate better ranking); VKA = vitamin k antagonist.

a Statistically significant *P* < 0.05.

### Antiplatelet strategies in special populations

Further analysis of specific patient populations was performed. In atrial fibrillation with concomitant stable CAD (Supplemental Table 3), 3 key studies (AFIRE, EPIC-CAD, and Lamberts cohort) consistently demonstrated that OAC monotherapy was superior to combination therapy in patients over 12 months from revascularization, with significant reductions in bleeding risk (30% to 68% relative risk reduction) without compromise in ischemic protection. In diabetic patients with stable CAD (Supplemental Table 4), ticagrelor + ASA showed modest benefit in the overall THEMIS population (HR 0.90 [0.81-0.99]; *P* value = 0.04) but significantly greater benefit in the THEMIS-PCI subgroup with prior PCI (HR 0.85 [0.74-0.97]; *P* = 0.013), with significant interaction by PCI status (*P* interaction = 0.043). Effect modifications by diabetes duration and HbA1c levels were not statistically significant.

### Heterogeneity assessment and GRADE evidence certainty

**Figure 4:**
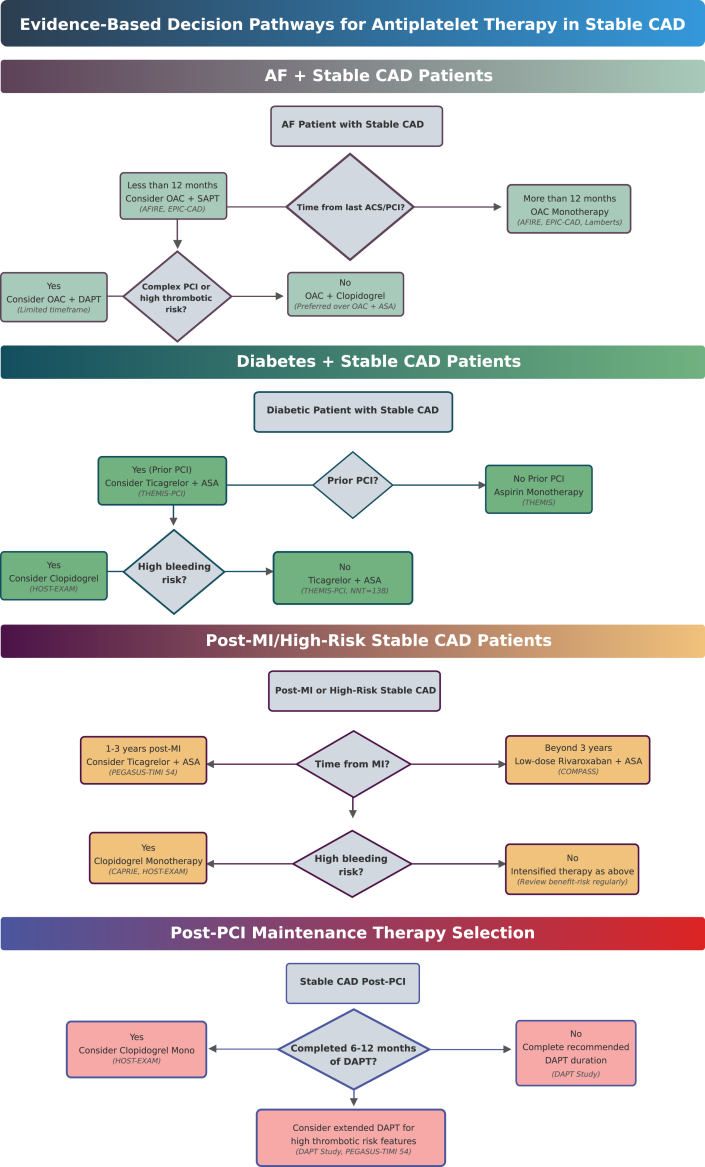
Evidence-Based Decision Pathways Roadmap for Stable Coronary Artery Disease

Heterogeneity assessment (Supplemental Table 4) revealed moderate heterogeneity for antiplatelet monotherapy comparisons (I^2^ = 43%) and OAC in atrial fibrillation with stable CAD patients (I^2^ = 28% for ischemic outcomes), but high heterogeneity for intensified therapy in high-risk stable CAD (I^2^ = 67%). Sensitivity analyses demonstrated improvement in heterogeneity with exclusion of CHARISMA (reduced to I^2^ = 42%). High between-group heterogeneity was observed for treatment effect by diabetes status (I^2^ = 51%; *P* = 0.04) and significant heterogeneity by prior MI status (I^2^ = 83%; *P* < 0.001), supporting differential treatment effects in specific patient subgroups. The GRADE assessment (Supplemental Table 5) demonstrated moderate certainty evidence for most efficacy comparisons, with high certainty evidence for intensified therapy bleeding risk and OAC monotherapy in atrial fibrillation with stable CAD population. Figure 4 presents the evidence-based decision pathways for antiplatelet therapy in different stable CAD subpopulations, integrating findings across all analyses.

Sensitivity analyses restricted to RCTs demonstrated consistent findings across all major comparisons. For the atrial fibrillation with stable CAD population, where the Lamberts cohort (n = 8,700) represented the only observational study among 3 included studies, RCT-only pooled estimates for efficacy (HR 0.61 [95% CI 0.49-0.76] from AFIRE and EPIC-CAD alone) were consistent with all-studies estimates (HR 0.70 [95% CI 0.59-0.84]), with overlapping 95% CIs. In a similar manner, for major bleeding, RCT-only estimates (HR 0.52 [95% CI 0.36-0.76]) aligned closely with all-studies estimates (HR 0.58 [95% CI 0.47-0.71]). Importantly, 3 of the 5 major treatment comparisons (clopidogrel vs aspirin, intensified antithrombotic therapy, and DAPT duration strategies) were based entirely on RCT evidence without any observational studies, confirming that core conclusions are mainly RCT-driven.

## Discussion

This systematic review and meta-analysis represent the most updated and extensive evidence synthesis to date on antiplatelet strategies for stable CAD. Our findings challenge several long-standing narratives and provide insights for therapeutic refinement across multiple patient populations. Three important messages are highlighted: First, clopidogrel monotherapy demonstrated superior efficacy and safety compared to aspirin in stable CAD patients; second, OAC monotherapy in atrial fibrillation with stable CAD patients conferred significant benefits over combination therapy; and third, intensified antithrombotic strategies showed selective benefit in specific high-risk subgroups, especially post-MI patients and diabetic patients with prior PCI (Central Illustration).

**Central Illustration:**
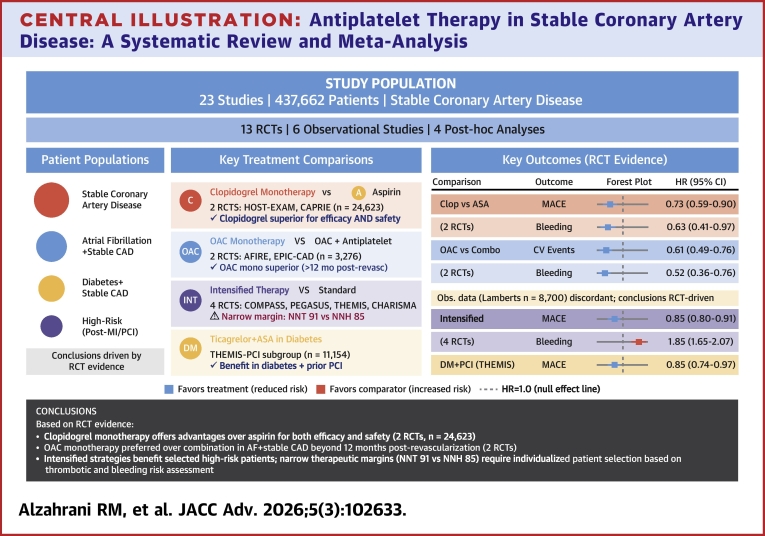
Antiplatelet Therapy in Stable Coronary Artery Disease: A Systematic Review and Meta-Analysis AF = atrial fibrillation; ASA = aspirin; CAD = coronary artery disease; Clop = clopidogrel; CV = cardiovascular; DM = diabetes mellitus; HR = hazard radio; MACE = major adverse cardiovascular events; MI = myocardial infarction; NNH = number needed to harm; NNT = number needed to treat; OAC = oral anticoagulant; Obs = observational; PCI = percutaneous coronary intervention; RCT = randomized controlled trial; SAPT = single antiplatelet therapy.

Several prior meta-analyses have investigated antiplatelet strategies in stable CAD populations,^13^, ^14^, ^15^, ^16^, ^17^, ^18^, ^19^, ^20^, ^21^, ^22^, ^23^ each addressing specific questions such as DAPT duration post-PCI,^17^^,^^18^ single-agent comparisons,^23^ or OAC strategies in atrial fibrillation with CAD.^15^^,^^16^^,^^22^ However, no prior analysis has comprehensively integrated evidence across all major clinical scenarios in a single framework. Our analysis extends beyond previous work in several critical ways. First, we include recently published landmark trials (HOST-EXAM 2021, EPIC-CAD 2024) that have advanced the evidence landscape. Second, we utilized network meta-analysis to enable indirect comparisons between treatments never directly compared. Third, we stratify findings across clinically distinct populations (atrial fibrillation with CAD, diabetes with CAD, post-MI high-risk) rather than pooling heterogeneous patients. Fourth, we calculate clinically meaningful NNT/NNH values across different baseline risk categories, facilitating individualized decision-making. Finally, we perform structured meta-regression to identify effect modifiers, revealing that treatment effects vary significantly by patient characteristics (age, diabetes status, prior MI) that were not evaluated in prior syntheses.

Compared to recent meta-analyses, Ahmed et al.^15^ investigated OAC monotherapy vs dual therapy in atrial fibrillation with CAD but included only 4 studies through 2023, missing the EPIC-CAD trial. Yuan et al.^23^ compared aspirin vs clopidogrel but included only CAPRIE and smaller studies, lacking HOST-EXAM's post-PCI evidence. Rashedi et al.^13^ focused on atrial fibrillation with CAD without addressing broader stable CAD populations or diabetes-specific strategies. Our integrated approach addresses these gaps while maintaining proper methodology including GRADE certainty assessments and risk-of-bias evaluation across different study designs.

A significant finding is the consistent superiority of clopidogrel over aspirin when monotherapy is indicated, based on direct head-to-head comparisons. While aspirin has formed the cornerstone of antithrombotic management in stable CAD for several decades, recent evidence from HOST-EXAM along with the historical CAPRIE trial demonstrates that clopidogrel monotherapy provides 27% relative risk reduction in composite cardiovascular endpoints with concurrent 37% reduction in major bleeding events. This favorable scenario of improved efficacy with improved safety stands in contrast to most antithrombotic strategy comparisons, where efficacy and safety usually demonstrate inverse relationships. Our network meta-analysis further supports this finding, with clopidogrel achieving the highest SUCRA ranking for combined efficacy-safety outcomes. These findings suggest that P2Y12 inhibition may offer advantages over aspirin for long-term thrombotic risk reduction in stable atherosclerotic disease.^48^

While our findings support clopidogrel's favorable profile in stable CAD, several practical considerations warrant mention. Clopidogrel's efficacy depends on CYP2C19-mediated hepatic activation, and carriers of loss-of-function alleles (affecting 25% to 30% of certain populations) may experience reduced drug effectiveness. However, routine pharmacogenomic testing remains Class IIb in current guidelines due to cost considerations and inconsistent evidence for outcome improvement across populations. The trials included in our analysis (HOST-EXAM, CAPRIE) did not routinely perform genotyping, suggesting that clopidogrel's benefits observed in these studies reflect real-world effectiveness across genetically heterogeneous populations. Recent developments in antiplatelet drug availability warrant consideration. Generic prasugrel has become available in many markets, and ticagrelor pricing has decreased substantially since its initial introduction. These changes may improve access to potent P2Y12 inhibitor-based strategies in settings where cost previously limited therapeutic options, potentially shifting the cost-effectiveness calculus for certain patient populations.^49^^,^^50^

It is important to note that our conclusions regarding clopidogrel's superiority are based on direct head-to-head comparisons with aspirin (HOST-EXAM, CAPRIE), in which ticagrelor was evaluated primarily in combination with aspirin rather than as monotherapy in stable CAD populations. The bleeding risk observed with ticagrelor plus aspirin combinations (HR 2.32 [1.82-2.94]) significantly offset the ischemic benefits (HR 0.90 [0.81-0.99]), resulting in narrow therapeutic margins. Direct comparisons between ticagrelor monotherapy and aspirin monotherapy are lacking in stable CAD populations, precluding definitive conclusions about ticagrelor's role as SAPT in this setting. The ONSET/OFFSET and STEEL-PCI studies demonstrated ticagrelor's superior pharmacodynamic profile but did not assess long-term clinical outcomes with monotherapy strategies.

The management of patients with concomitant atrial fibrillation and stable CAD represents another area where our findings provide important insights for practice. The traditional approach that such patients require combination therapy with OAC plus antiplatelet therapy has been challenged by recent evidence. Our findings reveal that OAC monotherapy not only provides noninferior protection against ischemic events but significantly reduces bleeding complications by 40% to 50% compared to combination regimens. This finding was consistent across studies, with AFIRE, EPIC-CAD, and the Lamberts cohort all demonstrating either noninferior or superior efficacy alongside improved safety with monotherapy strategies. The clinical implications include potential simplification of antithrombotic regimens in these patients, offering an opportunity to reduce bleeding risk without compromising thrombotic protection once patients are beyond 12 months from their most recent acute coronary event or revascularization procedure.

Regarding intensified antithrombotic therapy in high-risk stable CAD, our findings support a targeted approach rather than broad implementation. While the pooled estimates demonstrated little cardiovascular benefit with a 15% relative risk reduction, this comes at the expense of increased bleeding risk with 85% relative risk increase, resulting in a narrow therapeutic margin where NNT and NNH values are nearly equivalent in unselected populations. However, our meta-regression and subgroup analyses reveal important effect modifiers that may guide therapy selection. Patients with prior MI (PEGASUS-TIMI 54 trial), extensive atherosclerotic burden (COMPASS trial), or diabetes with prior PCI (THEMIS-PCI) demonstrated more favorable benefit-risk profiles with intensified therapy. It is important to distinguish between 2 fundamentally different comparisons when interpreting our findings. First, clopidogrel monotherapy vs aspirin monotherapy represents a comparison of single antiplatelet agents where clopidogrel demonstrated both superior efficacy and safety. Second, DAPT with clopidogrel plus aspirin vs aspirin monotherapy represents intensification of therapy, where the addition of clopidogrel to aspirin predictably increases bleeding risk while providing modest ischemic benefit. These are distinct clinical questions with different risk-benefit implications: The former supports clopidogrel as a potentially superior single agent, while the latter reflects the expected trade-off of combination therapy.^51^, ^52^, ^53^

The identification of diabetic patients with prior PCI as a population that may especially benefit from intensified therapy represents an important finding in stable CAD management. Our analysis of the THEMIS trial and its PCI subgroup revealed that ticagrelor addition to aspirin provided little benefit in the overall diabetic stable CAD population, with significantly greater benefit in those with prior PCI. This interaction by revascularization status suggests that the combined presence of diabetes and coronary stents identifies a patient subset with persistently heightened platelet reactivity and thrombotic risk. Importantly, this benefit extends well beyond the standard post-PCI timeframe, with median follow-up of 39.9 months in THEMIS, suggesting that certain patient subsets may benefit from sustained DAPT beyond currently recommended durations.

Our findings may inform clinical practice and guideline development. Current guidelines recommend aspirin monotherapy as the default strategy in stable CAD. The European Society of Cardiology guidelines acknowledge the possibility of alternatives to aspirin in selected patients, while American guidelines have maintained a more conservative stance. Our structured synthesis suggests that clopidogrel monotherapy may be considered as an option when antiplatelet monotherapy is indicated, particularly in patients with higher bleeding risk.^6^^,^^54^, ^55^, ^56^ For atrial fibrillation with stable CAD beyond 12 months from acute events, OAC monotherapy may be considered rather than combination therapy. For post-MI patients, especially with additional risk factors such as diabetes or polyvascular disease, extended dual pathway inhibition with either low-dose rivaroxaban or ticagrelor may be considered with regular reassessment of bleeding risk.

The evidence-based decision pathways we present (Figure 4) integrate these findings into practical algorithms that may inform clinical decision-making based on individual patient characteristics. The pathways address 4 common scenarios (atrial fibrillation with stable CAD, diabetes with stable CAD, post-MI/high-risk stable CAD, and post-PCI maintenance therapy) with considerations based on available evidence for each population. Implementation of these pathways may help inform care decisions while considering individual patient risk factors.

### Study Limitations

Several limitations warrant consideration. Heterogeneity in study populations, designs, and outcome definitions introduced complexity despite random-effects modeling and sensitivity analyses, particularly for intensified therapy (I^2^ = 67%). Some studies were open-label with methodological limitations, and changing definitions required endpoint harmonization. Few direct head-to-head comparisons necessitated network meta-analysis with transitivity assumptions. Individual patient data were unavailable, and generalizability to elderly patients (>85 years), advanced chronic kidney disease, and malignancy populations remains limited. Regarding observational study inclusion, only 2 comparisons meaningfully pooled observational with RCT data; critically, RCT-restricted sensitivity analyses demonstrated consistent estimates (atrial fibrillation with stable CAD: RCT-only HR 0.61 vs all-studies HR 0.70, overlapping CIs). Three of 5 major comparisons were based entirely on RCT evidence, confirming RCT-driven conclusions. Publication bias cannot be excluded, and the evolving antiplatelet landscape requires ongoing evidence synthesis.

## Conclusions

This systematic review and meta-analysis of 23 studies (437,662 patients) provide clinically relevant insights for antiplatelet therapy in stable CAD. Based on 2 RCTs, clopidogrel monotherapy demonstrated superior efficacy and safety compared to aspirin. In atrial fibrillation with stable CAD beyond 12 months post-revascularization, RCT evidence (AFIRE, EPIC-CAD) supports OAC monotherapy over combination therapy; however, observational data showed discordant results, reinforcing RCT-driven conclusions. Intensified antithrombotic strategies showed selective benefit in high-risk populations, particularly post-MI patients and diabetics with prior PCI; however, narrow therapeutic margins (NNT 91 vs NNH 85) necessitate individualized patient selection.

Perspectives **COMPETENCY IN MEDICAL KNOWLEDGE:** This meta-analysis synthesizes evidence from 23 studies demonstrating that among antiplatelet monotherapy options, 2 RCTs (HOST-EXAM, CAPRIE) showed clopidogrel superiority over aspirin for both efficacy and safety outcomes. In atrial fibrillation with stable CAD, RCT evidence supports OAC monotherapy over combination therapy beyond 12 months from revascularization. Intensified antithrombotic regimens provide modest ischemic benefit with significantly increased bleeding risk (NNT 91 vs NNH 85).

**COMPETENCY IN PATIENT CARE:** These findings may inform antiplatelet selection in stable CAD, particularly for patients at higher bleeding risk or those with aspirin intolerance. Clinicians should recognize that clopidogrel efficacy depends on CYP2C19-mediated activation, potentially affecting 25% to 30% of certain populations. Shared decision-making should incorporate individual bleeding risk, prior events, and patient preferences when selecting antithrombotic strategies.

**TRANSLATIONAL OUTLOOK:** Future research should evaluate clopidogrel effectiveness across different CYP2C19 phenotypes and racial/ethnic populations through pragmatic trials. Implementation studies examining real-world barriers to evidence-based antiplatelet adoption, including cost considerations with now-generic prasugrel and reduced ticagrelor pricing, may accelerate translation of these findings into clinical practice. Development of validated risk-prediction tools may help identify patients most likely to benefit from specific antithrombotic strategies.

## Funding support and author disclosures

The authors have reported that they have no relationships relevant to the contents of this paper to disclose.
