# Supplementary material for: Antiplatelet Therapy in Stable Coronary Artery Disease: A Systematic Review and Meta-Analysis
Source: JACC Adv. 2026 Mar 25;5(3):102633. doi: 10.1016/j.jacadv.2026.102633 (PMC13352007; doi:10.1016/j.jacadv.2026.102633)
Supplement: Supplemental Tables 1-5 [file mmc1.pdf]

Supplementary Table 1: Risk of Bias Assessment.

Study	Design	Randomization Process	Deviations from Intended Interventions	Missing Outcome Data	Measurement of Outcome	Selection of Reported Result	Overall Risk of Bias
**Randomized Controlled Trials (RoB 2)**							
HOST-EXAM (Koo et al., 2021)	RCT	Low	Some concerns[1]	Low	Some concerns[1]	Low	Some concerns
ASET Pilot (Kogame et al., 2020)	Single-arm pilot	N/A[2]	N/A[2]	Low	Low	Some concerns[3]	Some concerns
THEMIS (Steg et al., 2019)	RCT	Low	Low	Low	Low	Low	Low
EPIC-CAD (Cho et al., 2024)	RCT	Low	Some concerns[1]	Low	Low	Low	Some concerns
STEEL-PCI (Orme et al., 2018)	RCT	Some concerns[4]	Some concerns[1]	Low	Low	Low	Some concerns
COMPASS (Eikelboom et al., 2017)	RCT	Low	Low	Low	Low	Low	Low
PEGASUS-TIMI 54 (Bonaca et al., 2015)	RCT	Low	Low	Low	Low	Low	Low
ZEUS (Valgimigli et al., 2015)	RCT	Low	Some concerns[5]	Low	Low	Low	Some concerns
DAPT Study (Mauri et al., 2014)	RCT	Low	Low	Low	Low	Low	Low
ONSET/OFFSET (Gurbel et al., 2009)	RCT	Low	Low	Low	Low	Low	Low
CHARISMA (Bhatt et al., 2006)	RCT	Low	Low	Low	Low	Low	Low
CAPRIE (1996)	RCT	Some concerns[6]	Low	Low	Low	Low	Some concerns
AFIRE (Yasuda et al., 2019)	RCT	Low	Low	Low	Low	Low	Low
**Observational/Post-hoc Studies (ROBINS-I)**							
Öz et al., 2024 (DAPT-TR)	Observational	Serious[7]	Moderate	Low	Low	Low	Serious
Vallejo-Vaz et al., 2024 (RESRISK)	Retrospective cohort	Serious[7]	Low	Moderate[8]	Low	Low	Serious
Bian et al., 2024 (ATHENA-China)	Retrospective cohort	Serious[7]	Moderate	Moderate	Low	Low	Serious
Zou et al., 2023	Retrospective	Serious[7]	Moderate	Moderate	Low	Low	Serious
Sandner et al., 2022 (TiCAB Post-hoc)	Post-hoc analysis	Moderate	Low	Low	Low	Moderate[9]	Moderate
Pracoń et al., 2021 (ISCHEMIA-OMT)	Sub-analysis	Moderate	Low	Low	Low	Moderate[9]	Moderate
Leiter et al., 2021 (THEMIS-Diabetes)	Post-hoc analysis	Low	Low	Low	Low	Moderate[9]	Moderate
Lamberts et al., 2014	Nationwide cohort	Serious[7]	Moderate	Moderate	Low	Low	Serious
Bavry et al., 2015 (AJM)	Observational analysis	Serious[7]	Moderate	Moderate	Low	Low	Serious
Larsen et al., 2015 (PLOS ONE)	Cross-sectional	Moderate	Low	Low	Low	Low	Moderate

Abbreviations: *RCT = Randomized Controlled Trial; RoB 2 = Revised Cochrane Risk of Bias Tool for Randomized Trials; ROBINS-I = Risk Of Bias In Non-randomized Studies of Interventions; N/A = Not Applicable.*

Supplementary Table 2: OAC Strategies in AF + Stable CAD Patients.

Characteristic	AFIRE (2019)	EPIC-CAD (2024)	Lamberts et al. (2014)
**Study Design**	Multicenter, open-label, randomized trial	Multicenter, open-label, adjudicator-masked RCT	Nationwide cohort study (observational)
**Country**	Japan	South Korea	Denmark
**Sample Size**	2,236 (mITT: 2,215)	1,040	8,700
**Patient Population**	AF + Stable CAD (PCI/CABG >1yr OR angio confirmed CAD)	AF + Stable CAD (PCI/CABG ≥6mo or med managed confirmed CAD)	AF patients with stable CAD (≥12 months from MI or PCI)
**Mean Age (years)**	~74	72.1	74.2
**Male (%)**	~79%	77.1%	62%
**Prior MI (%)**	~35%	~16%	~64%
**Prior PCI (%)**	~70.6%	~60%	~39%
**Prior CABG (%)**	~11.4%	~7-8%	Not reported
**Diabetes (%)**	~42%	~40%	~17%
**Hypertension (%)**	~90%	~81%	~67%
**CHA_2DS_2-VASc Score**	Reported in full paper	4.3 ± 1.6 / 4 (3-5)	Not reported
**HAS-BLED Score**	Reported in full paper	2.1 ± 0.8 / 2 (2-3)	Not reported
**Intervention Arms**	1) Rivaroxaban Monotherapy (10-15mg daily, n=1,107)<br>2) Rivaroxaban + SAPT (n=1,108)	1) Edoxaban Monotherapy (n=524)<br>2) Edoxaban + SAPT (n=516)	Multiple regimens: VKA mono; VKA+ASA; VKA+clopidogrel; VKA+DAPT; ASA mono; Clopidogrel mono; ASA+clopidogrel
**SAPT Type in Combination Arm**	Aspirin: 70.2% P2Y12i (mostly clopidogrel): 26.8%	Aspirin or Clopidogrel (proportions not specified)	Various (primarily aspirin)
**Follow-up Duration**	Median 24.1 months (stopped early)	12 months	Mean 3.3 years
**Primary Efficacy Outcome Definition**	Composite: Stroke, systemic embolism, MI, unstable angina requiring revasc, or death from any cause	Net Adverse Clinical Events: Death, MI, stroke, systemic embolism, urgent revasc, OR major/CRNM bleeding	MI/coronary death; Thromboembolism (separate outcomes)
**Primary Efficacy Results**	Riva mono: 4.14%/yr vs Riva+SAPT: 5.75%/yr HR 0.72 (0.55-0.95) p=0.02 for superiority (post-hoc)	Edox mono: 6.8% vs Edox+SAPT: 16.2% HR 0.44 (0.30-0.65) p<0.001	MI/Coronary Death: VKA+ASA vs VKA mono: HR 1.12 (0.94-1.34) VKA+Clop vs VKA mono: HR 1.53 (0.93-2.52) Thromboembolism: No significant difference between VKA-containing regimens
**Major Bleeding Definition**	ISTH criteria	ISTH criteria	Serious bleeding (requiring hospitalization)
**Major Bleeding Results**	Riva mono: 1.62%/yr vs Riva+SAPT: 2.76%/yr HR 0.59 (0.39-0.89) p=0.01	Edox mono: 1.3% vs Edox+SAPT: 4.5% HR 0.32 (0.14-0.73)	VKA+ASA vs VKA mono: HR 1.50 (1.23-1.82) VKA+Clop vs VKA mono: HR 1.84 (1.11-3.06)
**All-Cause Mortality**	Riva mono: 1.85%/yr vs Riva+SAPT: 3.37%/yr HR 0.55 (0.38-0.81)	Edox mono: 0.6% vs Edox+SAPT: 0.7% HR 1.29 (0.29-5.76)	Not separately reported
**Cardiovascular Death**	Riva mono: 1.17%/yr vs Riva+SAPT: 1.99%/yr HR 0.59 (0.36-0.96)	Edox mono: 0.4% vs Edox+SAPT: 0.2% HR 1.66 (0.16-17.14)	Not separately reported
**Myocardial Infarction**	Riva mono: 0.59%/yr vs Riva+SAPT: 0.37%/yr HR 1.60 (0.67-3.87)	Edox mono: 0% vs Edox+SAPT: 0.5% NR (proportional hazards not met)	Included in MI/coronary death outcome
**Stroke (any)**	Ischemic Stroke: Riva mono: 0.96%/yr vs Riva+SAPT: 1.31%/yr HR 0.73 (0.42-1.29)	Edox mono: 1.4% vs Edox+SAPT: 0.8% NR (proportional hazards not met)	Included in thromboembolism outcome
**Combined Ischemic Events**	Major ischemic events reported separately	Edox mono: 1.6% vs Edox+SAPT: 1.8% HR 1.23 (0.48-3.10)	Not reported
**Net Clinical Benefit**	Not specifically reported but favorable for monotherapy	Strongly favors monotherapy (driven by reduced bleeding)	Combined endpoint (MI, thromboembolism, bleeding, all-cause death): VKA+ASA vs VKA mono HR 1.15 (1.03-1.29)
**Subgroup Analysis by Time from PCI/CABG**	Available in original publication	Not reported	Not reported
**Subgroup Analysis by SAPT Type**	Not reported	Not reported	VKA+ASA vs VKA mono and VKA+Clop vs VKA mono reported separately
**Early Termination**	Yes, due to increased mortality in combination arm	No	Not applicable (observational)
**Key Conclusion**	Rivaroxaban monotherapy was non-inferior and post-hoc superior for efficacy and superior for safety compared to combination therapy	Edoxaban monotherapy significantly reduced net adverse clinical events with similar ischemic outcomes and less bleeding	Addition of antiplatelet therapy to VKA in AF patients with stable CAD increased bleeding risk without reducing recurrent coronary events

***Abbreviations**: OAC = Oral Anticoagulant; AF = Atrial Fibrillation; Stable CAD = Stable Coronary Artery Disease; RCT = Randomized Controlled Trial; mITT = modified Intention-To-Treat; MI = Myocardial Infarction; PCI = Percutaneous Coronary Intervention; CABG = Coronary Artery Bypass Grafting; SAPT = Single Antiplatelet Therapy; DAPT = Dual Antiplatelet Therapy; VKA = Vitamin K Antagonist; ASA = Acetylsalicylic Acid (Aspirin); ISTH = International Society on Thrombosis and Haemostasis; CRNM = Clinically Relevant Non-Major; HR = Hazard Ratio; NR = Not Reported.*

Supplementary Table 3: Antiplatelet Strategies in Diabetes + Stable CAD Patients.

**Outcome Measure**	**THEMIS Overall (n=19,220)**	**THEMIS-PCI Subgroup (n=11,154)**	**ATHENA-China (n=509)**	**PEGASUS-TIMI 54 Diabetes Subgroup (n=~6,800)**	**CHARISMA Diabetes Subgroup (n=~6,500)**
**Primary Efficacy Outcome**	CV death, MI, stroke	CV death, MI, stroke	MACE (Cardiac death, MI, or stroke)	CV death, MI, stroke	CV death, MI, stroke
Event Rate - Intensified	736/9619 (7.7%)	404/5558 (7.3%)	29/509 (5.7%) real-world	T60: 338/2308 (14.7%)	277/3146 (8.8%)
Event Rate - Control	818/9601 (8.5%)	480/5596 (8.6%)	N/A (single arm)	414/2352 (17.6%)	299/3322 (9.0%)
Hazard/Risk Ratio (95% CI)	HR 0.90 (0.81-0.99)	HR 0.85 (0.74-0.97)	N/A (observational)	HR 0.82 (0.71-0.95)	RR 0.98 (0.84-1.14)
P-value	0.04	0.013	N/A	0.008	0.77
NNT (95% CI)	125 (65-1449)	77 (46-224)	N/A	35 (22-87)	500 (NNT to NNH 82)
**Major Bleeding**	TIMI Major	TIMI Major	BARC type ≥3	TIMI Major	GUSTO Severe
Event Rate - Intensified	206/9562 (2.2%)	111/5558 (2.0%)	8/509 (1.6%) real-world	T60: 92/2308 (4.0%)	67/3146 (2.1%)
Event Rate - Control	100/9531 (1.0%)	57/5596 (1.0%)	N/A (single arm)	25/2352 (1.1%)	50/3322 (1.5%)
Hazard/Risk Ratio (95% CI)	HR 2.32 (1.82-2.94)	HR 2.03 (1.48-2.76)	N/A (observational)	HR 3.91 (2.50-6.10)	RR 1.41 (0.98-2.03)
P-value	<0.001	<0.001	N/A	<0.001	0.06
NNH (95% CI)	93 (71-135)	100 (70-172)	N/A	35 (28-47)	167 (85-∞)
**Net Clinical Benefit**	Not calculated	Favorable	N/A	Neutral	Unfavorable
**Effect Modifiers (PCI Status):**					
Prior PCI - HR (95% CI)	0.85 (0.74-0.97)	N/A (all had PCI)	N/A	0.78 (0.65-0.93)	0.90 (0.72-1.13)
No Prior PCI - HR (95% CI)	0.95 (0.81-1.11)	N/A	N/A	0.90 (0.71-1.15)	1.04 (0.84-1.29)
P-interaction	0.043	N/A	N/A	0.31	0.31
**Effect Modifiers (Diabetes Duration):**					
≤5 years - HR (95% CI)	0.88 (0.71-1.09)	0.85 (0.64-1.12)	N/A	Not reported	Not reported
>5-10 years - HR (95% CI)	0.85 (0.70-1.03)	0.81 (0.62-1.05)	N/A	Not reported	Not reported
>10 years - HR (95% CI)	0.92 (0.80-1.06)	0.87 (0.72-1.04)	N/A	Not reported	Not reported
P-interaction	0.81	0.88	N/A	Not reported	Not reported
**Effect Modifiers (HbA1c):**					
≤7.0% - HR (95% CI)	0.86 (0.72-1.02)	0.82 (0.65-1.03)	N/A	Not reported	Not reported
>7.0-8.0% - HR (95% CI)	0.90 (0.73-1.09)	0.84 (0.65-1.09)	N/A	Not reported	Not reported
>8.0% - HR (95% CI)	0.95 (0.79-1.14)	0.89 (0.70-1.13)	N/A	Not reported	Not reported
P-interaction	0.61	0.82	N/A	Not reported	Not reported

Abbreviations: *Stable CAD = Stable Coronary Artery Disease; ASA = Acetylsalicylic Acid (Aspirin); BID = Twice Daily; CV = Cardiovascular; MI = Myocardial Infarction; HR = Hazard Ratio; RR = Risk Ratio; CI = Confidence Interval; NNT = Number Needed to Treat; NNH = Number Needed to Harm; PCI = Percutaneous Coronary Intervention; MACE = Major Adverse Cardiovascular Events; TIMI = Thrombolysis In Myocardial Infarction; ISTH = International Society on Thrombosis and Haemostasis; GUSTO = Global Utilization of Streptokinase and Tissue Plasminogen Activator for Occluded Coronary Arteries; BARC = Bleeding Academic Research Consortium; N/A = Not Applicable or Not Available.*

Supplementary Table 4: Heterogeneity Assessment of Key Treatment Comparisons.

**Treatment Comparison**	**Statistical Heterogeneity**	**Clinical Heterogeneity Sources**	**Sensitivity Analysis Results**
Antiplatelet Monotherapy:			
Clopidogrel vs. Aspirin	Primary efficacy outcome: I^2 = 43%, p = 0.18	• Study era (1996 vs. 2021)<br>• Population (post-PCI vs. mixed atherosclerotic)<br>• Aspirin dose (325mg vs. 100mg)<br>• Background therapy	Not performed due to limited studies
OAC in AF + Stable CAD:			
OAC mono vs. OAC+SAPT	Ischemic outcomes: I^2 = 28%, p = 0.25<br>Bleeding outcomes: I^2 = 58%, p = 0.09	• OAC type (VKA vs. DOAC)<br>• SAPT type (aspirin vs. P2Y12i)<br>• Time from last revascularization<br>• Patient age	Exclusion of observational data (Lamberts):<br>• Efficacy: HR 0.69 [0.57-0.84]<br>• Bleeding: HR 0.52 [0.38-0.71]
Intensified Therapy in High-Risk Stable CAD:			
DAPT/Riva+ASA vs. ASA	MACE outcomes: I^2 = 67%, p = 0.03<br>Bleeding outcomes: I^2 = 76%, p = 0.006	• Intensification strategy<br>• Baseline risk profile<br>• Prior MI percentage<br>• Diabetes prevalence<br>• Study duration<br>• Primary endpoint components	Exclusion of CHARISMA:<br>• MACE: I^2 reduced to 42%, p = 0.18<br>• HR 0.83 [0.77-0.89]<br><br>Subgroup by strategy type:<br>• Ticagrelor+ASA: I^2 = 28%; Riva+ASA vs. Clop+ASA: I^2 = 64%
Ticagrelor+ASA vs. ASA in DM + Stable CAD:			
Primary analysis	Primary efficacy outcome: I^2 = 18%, p = 0.27<br>Major bleeding: I^2 = 5%, p = 0.31	• Study design (RCT vs. observational)<br>• PCI status<br>• Diabetes duration/control<br>• Ticagrelor dose (90mg→60mg)	Exclusion of observational data:<br>• No significant change in effect estimates
DAPT Duration Strategies:			
Extended vs. Standard DAPT	Stent thrombosis: I^2 = 72%, p = 0.06<br>Major bleeding: I^2 = 82%, p = 0.02	• Different duration comparisons<br>• Stent types<br>• P2Y12i used (clopidogrel vs. prasugrel)<br>• Baseline risk	Not performed due to limited studies and different designs
Between-Group Heterogeneity			
Treatment effect by patient subgroups	Prior MI/no MI: I^2 = 83%, $p < 0.001$<br>Diabetes/no diabetes: I^2 = 51%, p = 0.04<br>CKD/no CKD: I^2 = 70%, p = 0.03<br>Age ≥75/<75: I^2 = 35%, p = 0.16	• Risk factor interactions<br>• Different treatment strategies<br>• Background therapy	Meta-regression by baseline risk:<br>• Higher baseline risk associated with greater relative benefit (p = 0.03)<br>• Greater absolute benefit (p = 0.01)<br>• Also greater absolute harm (p = 0.02)

Abbreviations: *ASA = Aspirin; CI = Confidence Interval; CKD = Chronic Kidney Disease; Clop = Clopidogrel; DAPT = Dual Antiplatelet Therapy; DM = Diabetes Mellitus; DOAC = Direct Oral Anticoagulant; HR = Hazard Ratio;* I^2 *= I-squared statistic (measure of statistical heterogeneity); MACE = Major Adverse Cardiovascular Events; MI = Myocardial Infarction; OAC = Oral Anticoagulant; PCI = Percutaneous Coronary Intervention; P2Y12i = P2Y12 Inhibitor; Riva = Rivaroxaban; SAPT = Single Antiplatelet Therapy; Stable CAD = Stable Coronary Artery Disease; Tica = Ticagrelor; VKA = Vitamin K Antagonist.*

Supplementary Table 5: GRADE Summary of Findings.

Clinical Question	Outcome	Relative Effect (95% CI)	Quality of Evidence	Reasons for Rating	Absolute Effect
Clopidogrel vs. Aspirin Monotherapy in Stable CAD:					
Does clopidogrel monotherapy provide superior outcomes to aspirin in Stable CAD?	MACE	RR 0.79 (0.71-0.87)	⊕⊕⊕⊖ Moderate	Downgraded for inconsistency	14 fewer events per 1000 (9-19)
	Stroke	RR 0.64 (0.51-0.81)	⊕⊕⊕⊖ Moderate	Downgraded for inconsistency	6 fewer events per 1000 (3-8)
	MI	RR 0.85 (0.74-0.97)	⊕⊕⊕⊖ Moderate	Downgraded for inconsistency	4 fewer events per 1000 (1-7)
	Major Bleeding	RR 0.87 (0.68-1.11)	⊕⊕⊖⊖ Low	Downgraded for inconsistency and imprecision	1 fewer event per 1000 (3 fewer to 1 more)
OAC Strategies in AF + Stable CAD:					
Is OAC monotherapy non-inferior to OAC+SAPT in AF + Stable CAD?	MACE/Composite Efficacy	HR 0.70 (0.59-0.84)	⊕⊕⊕⊕ High	Large effect	18 fewer events per 1000 (10-25)
	Major Bleeding	HR 0.58 (0.47-0.71)	⊕⊕⊕⊕ High	Large effect and consistency	12 fewer events per 1000 (8-15)
	All-cause Mortality	HR 0.67 (0.53-0.85)	⊕⊕⊕⊖ Moderate	Upgraded for large effect, downgraded for inconsistency	11 fewer deaths per 1000 (5-16)
Intensified Antithrombotic Therapy in High-Risk Stable CAD:					
Does intensified antithrombotic therapy improve outcomes in high-risk Stable CAD?	MACE	HR 0.85 (0.80-0.90)	⊕⊕⊕⊖ Moderate	Downgraded for inconsistency	11 fewer events per 1000 (7-14)
	Major Bleeding	HR 1.73 (1.54-1.94)	⊕⊕⊕⊕ High	Consistency and precision	7 more events per 1000 (5-9)
	Cardiovascular Death	HR 0.91 (0.83-1.00)	⊕⊕⊖⊖ Low	Downgraded for inconsistency and imprecision	2 fewer deaths per 1000 (0-4)
Antiplatelet Strategies in Diabetes + Stable CAD:					
Do intensified antiplatelet strategies benefit patients with diabetes and Stable CAD?	MACE	HR 0.88 (0.80-0.97)	⊕⊕⊕⊖ Moderate	Downgraded for indirectness	9 fewer events per 1000 (2-15)
	Major Bleeding	HR 2.20 (1.73-2.79)	⊕⊕⊕⊖ Moderate	Downgraded for indirectness	12 more events per 1000 (7-18)
	MACE in Post-PCI	HR 0.85 (0.74-0.97)	⊕⊕⊕⊖ Moderate	Downgraded for imprecision	11 fewer events per 1000 (2-19)
	Major Bleeding in Post-PCI	HR 2.03 (1.48-2.76)	⊕⊕⊕⊖ Moderate	Downgraded for imprecision	10 more events per 1000 (5-18)

Abbreviations: *MACE = Major Adverse Cardiovascular Events; RCT = Randomized Controlled Trial; CI = Confidence Interval; RR = Risk Ratio; HR = Hazard Ratio; OAC = Oral Anticoagulant; SAPT = Single Antiplatelet Therapy; Stable CAD = Stable Coronary Artery Disease; AF = Atrial Fibrillation; MI = Myocardial Infarction; PCI = Percutaneous Coronary Intervention.*
